# The Role of Pyrophosphate in the Origin of Life: An Energy-Based Hypothesis

**DOI:** 10.3390/biology15181592

**Published:** 2026-09-09

**Authors:** Ricardo Villa-Bellosta

**Affiliations:** Department of Biochemistry and Molecular Biology, University of Santiago de Compostela, 15782 Santiago de Compostela, Spain; ricardo.villa@usc.es

**Keywords:** origin of life, inorganic pyrophosphate, prebiotic chemistry, bioenergetics, phosphate activation, non-enzymatic metabolism, chemical evolution, ATP evolution

## Abstract

Life could not emerge through molecular synthesis alone; it also required mechanisms that coupled environmental energy to directional chemical change. Yet, ATP, the dominant energy carrier in modern cells, depends on nucleotide synthesis, selective catalysis, and membrane-based regeneration, making it an unlikely starting point for bioenergetics. Here, I examine inorganic pyrophosphate as a possible intermediate between geochemical phosphate activation and ATP-centred metabolism. Condensed phosphates can form under plausible volcanic, mineral-mediated, and wet–dry conditions, and pyrophosphate has sufficient transfer potential to support selected phosphorylation reactions and ion translocation when appropriately coupled. I propose that recurrent pyrophosphate production could have promoted the formation of phosphorylated sugars, linking prebiotic carbon synthesis to non-enzymatic reaction networks resembling central metabolism. Activated enol and acyl phosphates may subsequently have regenerated pyrophosphate, allowing partial energetic closure. I further consider how polyphosphorylated pentoses could have provided transitional group-transfer scaffolds from which nucleotide-based activation chemistry emerged. In this view, ATP did not replace an unrelated primordial currency, but consolidated functions already distributed across simpler phosphate compounds. The persistence of pyrophosphate in biosynthetic directionality, phosphoryl transfer, and membrane energetics may therefore reflect a remnant of an earlier energetic architecture.

## Simple Summary

Life requires not only the formation of organic molecules but also a way to capture energy and use it to favour chemical reactions. Modern cells rely mainly on adenosine triphosphate for this purpose, but this complex molecule probably appeared after simpler forms of energy coupling. This article examines whether inorganic pyrophosphate, a simple compound made of two linked phosphate groups, could have contributed to this earlier stage. Experimental studies show that pyrophosphate can form under conditions relevant to the early Earth and can participate in phosphate transfer and other energy-dependent reactions. I propose that its recurrent local production could have helped connect early carbon chemistry with simple reaction networks and contributed to the gradual emergence of more complex energy systems. The model does not explain the origin of life as a whole, but identifies specific chemical transitions that can be tested experimentally.

## 1. Introduction

The origin of life was not simply a problem of molecular synthesis. It was also a problem of energy flow [1,2,3,4,5]. For prebiotic chemistry to cross the threshold towards biology, it had to do more than produce a variety of organic compounds [6,7]. Reactions had to become coupled in ways that imposed directionality, sustained chemical disequilibria, and allowed complexity to accumulate. Without such energetic organization, prebiotic chemistry would probably have remained largely transient: a collection of local reactions unable to support the persistent, self-reinforcing networks that define metabolism.

In modern biology, this role is dominated by adenosine triphosphate (ATP), the canonical energy currency of the cell [8,9]. ATP hydrolysis drives biosynthesis, transport, motility, signalling, and many other cellular processes. However, ATP is a structurally elaborate molecule, and its synthesis, regeneration, and use are embedded within enzyme-catalysed pathways, nucleotide metabolism, and membrane-associated systems of energy conservation. Its emergence as a universal energy currency therefore probably required a degree of molecular and metabolic organization that was absent from the earliest prebiotic systems [8,10].

This raises a central question in origins-of-life research: what form of energy coupling preceded the ATP-based economy? Any plausible pre-ATP energy mediator would need to satisfy several constraints. It would have to be chemically simple, accessible under prebiotic conditions, and capable of coupling exergonic chemistry to otherwise endergonic transformations. It would also need to function in environments lacking catalytic precision and compartmental regulation of modern cells.

Inorganic pyrophosphate satisfies several of these criteria [11]. Composed of two phosphate groups joined by a phosphoanhydride bond, pyrophosphate contains the same general class of phosphate-anhydride linkage found in ATP, but without the structural complexity of a nucleotide scaffold [12,13]. Experimental studies show that condensed phosphates can form under plausible volcanic and wet–dry conditions, and that pyrophosphate can mediate phosphate transfer to organic substrates under selected prebiotic conditions [14,15,16]. The free energy associated with its phosphoanhydride bond can also be harnessed, when appropriately coupled, for phosphoryl transfer [17,18] and ion transport [19,20]. These properties make pyrophosphate a plausible participant in early energy coupling, although its proposed role as a primordial energy currency remains debated [21].

The relevance of pyrophosphate does not rest solely on its prebiotic accessibility. In modern metabolism, pyrophosphate formation, hydrolysis, and transfer contribute to biosynthetic directionality, phosphoryl transfer, and ion-coupled energy transduction [18,20,21]. These conserved functions establish its chemical versatility, but do not by themselves demonstrate that equivalent roles preceded the emergence of modern metabolism [21,22].

In this manuscript, I review the available evidence and develop the hypothesis that inorganic pyrophosphate contributed to early bioenergetic organization as a local and recurrent energy-coupling intermediate between geochemical phosphate activation and ATP-centred metabolism [8,14,21,22]. I integrate evidence from geochemistry, thermodynamics, prebiotic chemistry, non-enzymatic metabolism, and modern biochemistry to identify which transitions are experimentally demonstrated, indirectly supported, or still hypothetical. The stages proposed below should be understood as functionally distinguishable and experimentally testable transitions, rather than as a unique or obligatory historical sequence.

Throughout this manuscript, “directionality” refers strictly to a kinetic or thermodynamic bias in reaction flux, such as suppression of reverse reactions or preferential accumulation of products, and does not imply teleological guidance or an intrinsic tendency of prebiotic chemistry towards biological function. The model addresses the energetic coupling and chemical persistence of selected reaction networks; it does not attempt to explain the origin of genetic information, heredity, or the integrated regulatory organization characteristic of living systems.

## 2. Chemical and Energetic Foundations of a Pre-ATP Role for Pyrophosphate

### 2.1. The Energetic Problem Before ATP

Before oxidative phosphorylation, photosynthesis, and enzyme-catalysed metabolism, chemical systems on the early Earth faced a fundamental constraint: environmental disequilibria had to be converted into chemical work without the molecular machinery of modern cells. Thermal and redox gradients, mineral interfaces, and fluctuating hydration conditions could all have supplied free energy, but only where that energy became coupled to chemical transformations and allowed products to accumulate. Alkaline hydrothermal systems illustrate one possible setting. Laboratory experiments have reproduced porous Fe(Ni)S-rich precipitates, thermal gradients, and continuously flowing interfaces that could have localized reactants and organized geochemical disequilibria before the emergence of biological membranes [5,23].

ATP is unlikely to have provided the earliest solution to this problem. Its synthesis and sustained use require nucleotides, selective catalysts, and mechanisms for continual regeneration. In modern cells, ATP regeneration is largely coupled to ion gradients across biological membranes through chemiosmotic phosphorylation [24,25]. ATP-based bioenergetics therefore presupposes a degree of molecular and cellular organization that is difficult to place at the outset of chemical evolution [8,10]. Simpler phosphorylated intermediates may consequently have preceded the establishment of ATP as the dominant cellular energy carrier [2].

### 2.2. Prebiotic Formation and Local Availability of Condensed Phosphates

The chemical properties of pyrophosphate make it a plausible coupling intermediate, but its prebiotic relevance would have depended on recurrent production and local availability [13,22,26]. Several experimentally supported routes could have generated condensed phosphate species on the early Earth. Heating ammonium dihydrogen phosphate with urea at 72–100 °C produced linear condensed phosphate chains and, under selected conditions, trimetaphosphate [27]. Volcanic activity offers a distinct route: experiments simulating magmatic conditions, together with analyses of volcanic condensates, showed that partial hydrolysis of P_4_O_10_ can yield water-soluble condensed phosphates [14]. More direct evidence for pyrophosphate formation is provided by the oxidation of reduced phosphorus species, which generates condensed phosphates including pyrophosphate [28]. Keefe and Miller likewise reported pyrophosphate formation from orthophosphate in several potentially prebiotic systems, including ammonium formate, thiocyanate oxidation, and polymerizing hydrogen cyanide [29].

**Figure 1:**
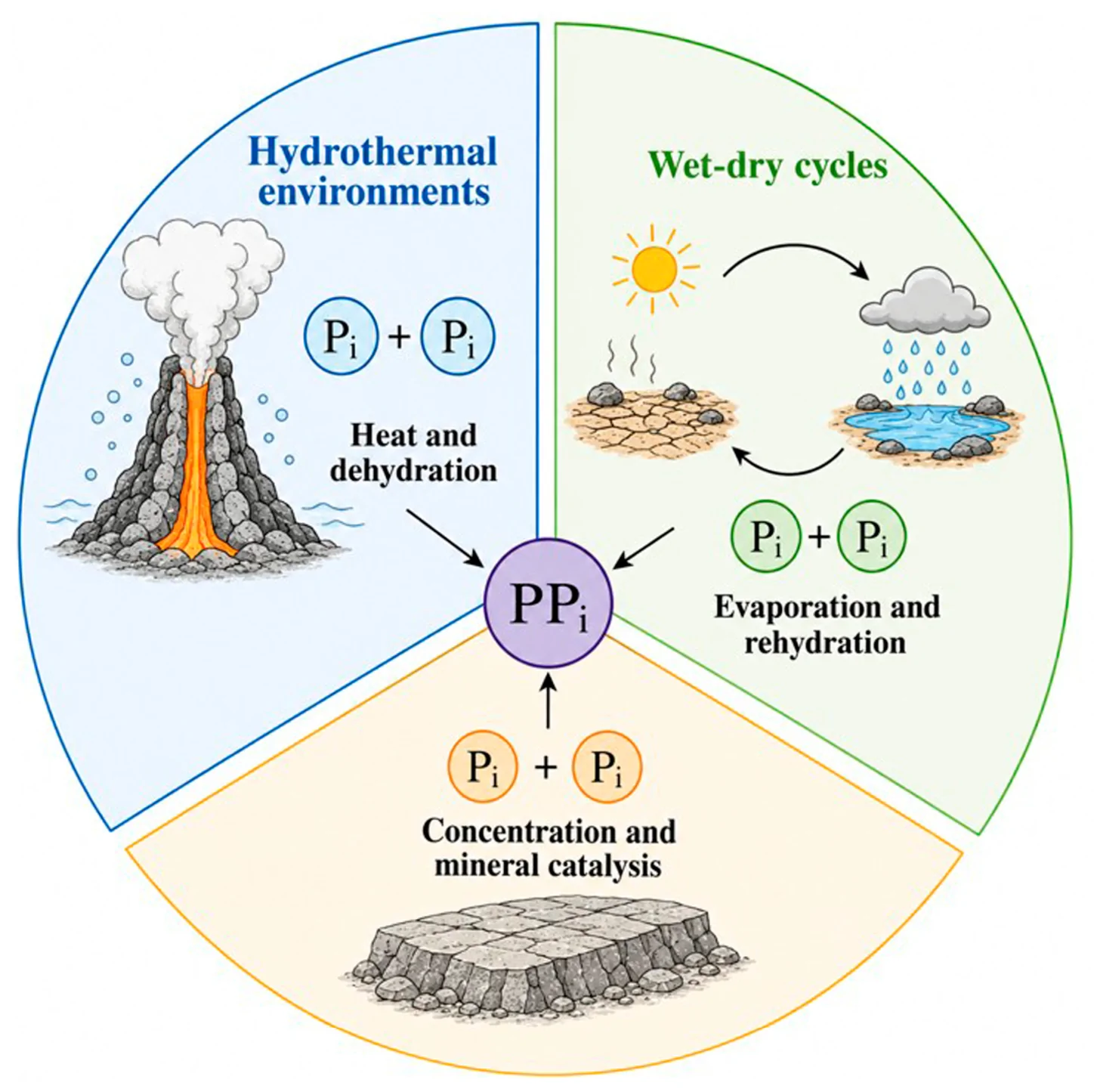
Proposed prebiotic routes to inorganic pyrophosphate formation. Orthophosphate (P_i_) could have condensed to pyrophosphate in hydrothermal settings, on mineral surfaces, and during wet–dry cycles. These environments provide complementary mechanisms of heating, dehydration, concentration, and surface catalysis that may have supported recurrent local production of condensed phosphates on the early Earth.

Mineral surfaces may have further organized this chemistry by adsorbing reactants and accelerating condensation reactions (Figure 1). In experiments with amorphous silica, deposited orthophosphate formed condensed phosphate species at considerably lower temperatures than bulk KH_2_PO_4_ [30]. Heating adsorbed AMP produced adenosine, ADP, and ATP through dismutation, whereas co-adsorption of AMP and orthophosphate at relatively high surface loadings allowed both AMP dismutation and phosphorylation by inorganic phosphate to contribute to ADP and ATP formation [30]. Drying shifted the equilibrium of phosphate condensation towards the products by lowering water activity, while the silica surface exerted primarily a kinetic effect, reducing the temperature required for condensation rather than substantially altering its thermodynamic driving force [31]. Mineral interfaces could therefore have provided localized settings in which phosphate species were adsorbed, concentrated during drying, and kinetically activated by heating.

Wet–dry cycling offers a complementary setting for phosphate condensation and transfer (Figure 1). Evaporation concentrates solutes and lowers water activity, favouring condensation, whereas limited rehydration restores molecular mobility and permits further reaction. Deliquescent salts can regulate this cycling by absorbing atmospheric water. In glycine-containing systems, deliquescent salt mixtures underwent repeated drying and spontaneous rehydration and produced substantially higher oligomerization yields than corresponding non-deliquescent controls [32]. Although these experiments did not examine phosphate chemistry directly, they demonstrate a mechanism by which wet–dry cycling could occur without regular rainfall. In a separate drying-pool model at 60–75 °C, pyrophosphate phosphorylated uridine to 5′-, 2′-, and 3′-uridine monophosphates, cyclic 2′,3′-uridine monophosphate, and a uridine–phosphate–uridine dimer [16]. Phosphorylation occurred without additives, but the highest organophosphate yields were obtained when quartz sand, Mg^2+^, and urea were present together. Fluctuating terrestrial environments could therefore have combined solute concentration, limited atmospheric rehydration, and phosphate transfer, although their integration within a single prebiotic reaction system remains to be demonstrated.

The relevant scale may therefore have been local rather than planetary. Pyrophosphate need not have accumulated globally or persisted indefinitely. Volcanic and geochemically reactive environments could have generated localized supplies of pyrophosphate and related condensed phosphate species [14,28,29], whereas drying cycles and mineral interfaces could have concentrated these compounds and increased their contact with organic substrates [16,30,31,32]. This interpretation is consistent with the conclusions of Keefe and Miller, who considered their condensed-phosphate syntheses insufficiently robust to have been important in the primitive open ocean but potentially applicable in localized environments where orthophosphate and condensing agents could become concentrated [29]. The proposed prebiotic relevance of pyrophosphate therefore depends less on global accumulation than on recurrent local production or concentration and on the productive capture of its transfer potential before hydrolysis.

### 2.3. Thermodynamics of Pyrophosphate and ATP Hydrolysis

**Figure 2:**
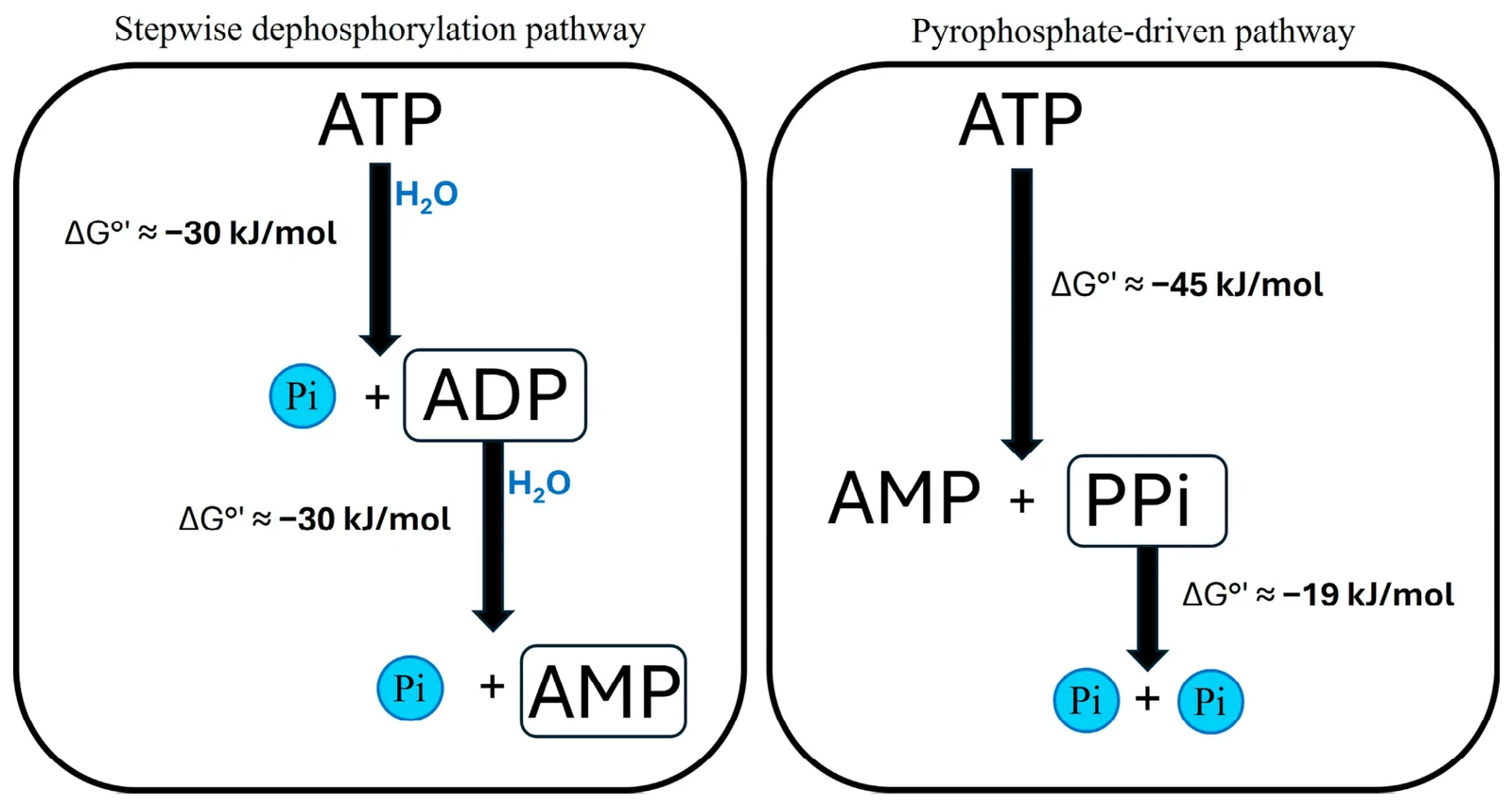
Thermodynamic consequences of alternative ATP cleavage routes. ATP hydrolysis to ADP and orthophosphate (P_i_) releases approximately −30 kJ mol^−1^, whereas cleavage to AMP and pyrophosphate (PP_i_) provides greater thermodynamic leverage. Subsequent PP_i_ hydrolysis further suppresses the reverse reaction and favours product formation. Values are approximate and depend on pH, Mg^2+^ concentration, ionic strength, and chemical speciation.

A common objection to an early bioenergetic role for pyrophosphate is whether its hydrolysis could have supplied sufficient free energy to support useful chemistry. The comparison with ATP, however, cannot be reduced to a pair of textbook values. The transformed standard free energy of either reaction depends on pH, Mg^2+^ binding, ionic strength, and the protonation states used to define the reaction, whereas the free energy available in a given system also depends on the activities of substrates and products [13,33,34]. Experimental measurements and thermodynamic calculations nevertheless show that both reactions are exergonic (Figure 2). Near neutral pH, reported standard transformed free energies are approximately −30.5 kJ mol^−1^ for ATP hydrolysis and approximately −19 kJ mol^−1^ for pyrophosphate hydrolysis [13,33,34,35].

The relevant question is therefore not whether pyrophosphate releases as much free energy as ATP under every set of conditions, but whether its consumption can be coupled to useful chemical or electrochemical work. In extant metabolism, pyrophosphate supports direct phosphoryl transfer [18,26] and drives H^+^ or Na^+^ translocation across biological membranes [36,37]. These reactions demonstrate its capacity for chemical and electrochemical coupling, although they do not imply that equivalent catalysts existed before the emergence of enzymes.

Pyrophosphate plays a distinct role in many biosynthetic reactions. Cleavage of ATP to AMP and pyrophosphate provides greater thermodynamic leverage than cleavage to ADP and orthophosphate (Figure 2), with reported standard transformed free-energy changes of approximately −45 kJ mol^−1^ [34,38]. In these reactions, ATP is not simply hydrolysed: its adenylyl group is transferred to a substrate, generating a reactive intermediate while releasing pyrophosphate. Subsequent hydrolysis of pyrophosphate adds a further exergonic step, suppresses the reverse reaction, and reinforces biosynthetic commitment [21]. This strategy is used in reactions requiring strong substrate activation, including amino acid adenylation and numerous cofactor and metabolite syntheses [39,40]. In nucleic acid polymerization, incorporation of the incoming nucleoside triphosphate releases pyrophosphate directly, and its subsequent hydrolysis limits pyrophosphorolysis and reinforces forward synthesis [41].

This distinction is central to prebiotic chemistry. Pyrophosphate hydrolysis could have favoured condensation or phosphate-transfer reactions only where its consumption was chemically linked to product formation. Direct phosphoryl transfer provides one possible route, whereas removal of pyrophosphate formed within a reaction sequence could suppress reverse flux and reinforce forward synthesis. Thermodynamics alone therefore neither establishes nor excludes an early bioenergetic role for pyrophosphate. Such a role would also have required recurrent production or regeneration and a means of coupling pyrophosphate turnover to productive chemistry [14,28]. Pyrophosphate is thus better viewed not as a weaker analogue of ATP, but as a distinct coupling intermediate capable of contributing to phosphoryl transfer, reaction directionality, and ion translocation [18,26,36,37,41].

### 2.4. What Pyrophosphate Can and Cannot Phosphorylate

#### 2.4.1. Terminal Versus Anomeric Phosphorylation

The thermodynamic data in Table 1 reveal a clear distinction between phosphorylation of terminal hydroxyl groups and phosphorylation at the anomeric carbon. Hydrolysis of ribose 5-phosphate has an estimated standard Gibbs energy change of approximately −15 kJ mol^−1^, based on the thermodynamic analysis of sugar-phosphate hydrolysis reported by Tewari and colleagues [42]. By contrast, hydrolysis of α-D-ribose 1-phosphate releases −22.6 ± 0.6 kJ mol^−1^ under standard biochemical conditions [43]. A closely related pattern is observed for glucose phosphates. Hydrolysis of glucose 6-phosphate has a standard Gibbs energy change of −13.72 ± 0.75 kJ mol^−1^ [44], whereas the value inferred for glucose 1-phosphate is close to −21 kJ mol^−1^ [44,45]. Moreover, the standard Gibbs energy of hydrolysis is −13.70 ± 0.28 kJ mol^−1^ for fructose 6-phosphate at 25 °C [42], whereas the older value reported for fructose 1-phosphate is approximately −10.3 kJ mol^−1^ at pH 8.5 and 38 °C [46,47].

In ribose and glucose, phosphorylation at the anomeric carbon produces a bond that is more activated than phosphorylation of the terminal primary alcohol, by about 7–8 kJ mol^−1^ [42,43,44,45]. Fructose is different: its anomeric carbon is C2, whereas C1 and C6 are both primary alcohols. Therefore, fructose 1-phosphate and fructose 6-phosphate behave as ordinary phosphate monoesters, consistent with their lower hydrolysis energies [42,46,47].

This thermodynamic distinction becomes especially relevant when pyrophosphate is considered as a phosphoryl donor. Under biochemical reference conditions, pyrophosphate hydrolysis releases approximately 17–20 kJ mol^−1^, although the exact value depends strongly on pH, ionic strength, and metal-ion concentration [13]. Coupling this reaction to the formation of ribose 5-phosphate, glucose 6-phosphate, or fructose 6-phosphate would therefore be only modestly exergonic, but still thermodynamically accessible (approximately −2 to −6 kJ mol^−1^ under the reference conditions considered). However, the same energetic margin is not available for phosphorylation at the anomeric C1 of aldoses. Based on the measured hydrolysis free energy of α-D-ribose 1-phosphate, its formation from ribose and pyrophosphate would be close to thermoneutral or slightly endergonic (approximately +3 to +6 kJ mol^−1^ under the reference conditions considered) [43]. Glucose 1-phosphate presents a similar case, given its inferred hydrolysis free energy of approximately −21 kJ mol^−1^ [44,45].

Pyrophosphate could therefore support phosphorylation of terminal primary alcohols more readily than direct anomeric activation. Accumulation of C1 phosphates would require an additional source of thermodynamic bias, such as dehydration, mineral-surface activation, an elevated pyrophosphate/phosphate ratio, or rapid removal of the product through a subsequent reaction. Pyrophosphate is therefore better suited to the formation of relatively low-energy terminal phosphate monoesters than to direct activation of the anomeric carbon of aldoses.

#### 2.4.2. PRPP as an Energetic Boundary Case

Two reactions in current metabolism illustrate the limits and possibilities of this chemistry. The first is the synthesis of phosphoribosyl pyrophosphate (PRPP), in which activation of the anomeric C1 requires ATP: ribose 5-phosphate + ATP → PRPP + AMP. PRPP synthetase (EC 2.7.6.1.) transfers the β,γ-diphosphoryl group of ATP directly to ribose 5-phosphate, rather than forming free pyrophosphate as an intermediate [49,50]. Cleavage of ATP to AMP provides greater thermodynamic leverage than cleavage to ADP and phosphate (Figure 2), consistent with the strongly activated nature of the anomeric diphosphate bond in PRPP. Indeed, hydrolysis of PRPP to ribose 5-phosphate and pyrophosphate has an estimated standard Gibbs energy change of approximately −29.3 ± 2.1 kJ mol^−1^ [48], making the reverse condensation of ribose 5-phosphate with free pyrophosphate strongly unfavourable in aqueous solution. PRPP synthesis therefore represents a distinct form of anomeric activation that requires direct transfer from a more strongly activated donor, rather than simple coupling to pyrophosphate hydrolysis.

#### 2.4.3. A Biochemical Precedent for Pyrophosphate-Dependent Sugar Phosphorylation

A contrasting case is provided by pyrophosphate-dependent phosphofructokinase (EC 2.7.1.90.), which catalyses the reversible reaction: fructose 6-phosphate + pyrophosphate ⇌ fructose 1,6-bisphosphate + phosphate. Here, pyrophosphate acts directly as the phosphoryl donor to the primary C1 hydroxyl of fructose 6-phosphate [17,26]. Because C1 of fructose is not anomeric, the product contains a conventional phosphate monoester. Therefore, this enzyme provides a direct biochemical precedent for coupling pyrophosphate consumption to sugar phosphorylation. Its reversibility also allows phosphate-bond energy to be conserved as pyrophosphate when fructose 1,6-bisphosphate reacts with phosphate.

## 3. Entry of Pyrophosphate into Primitive Carbon Chemistry

### 3.1. Formation and Selection of Prebiotic Sugars

**Figure 3:**
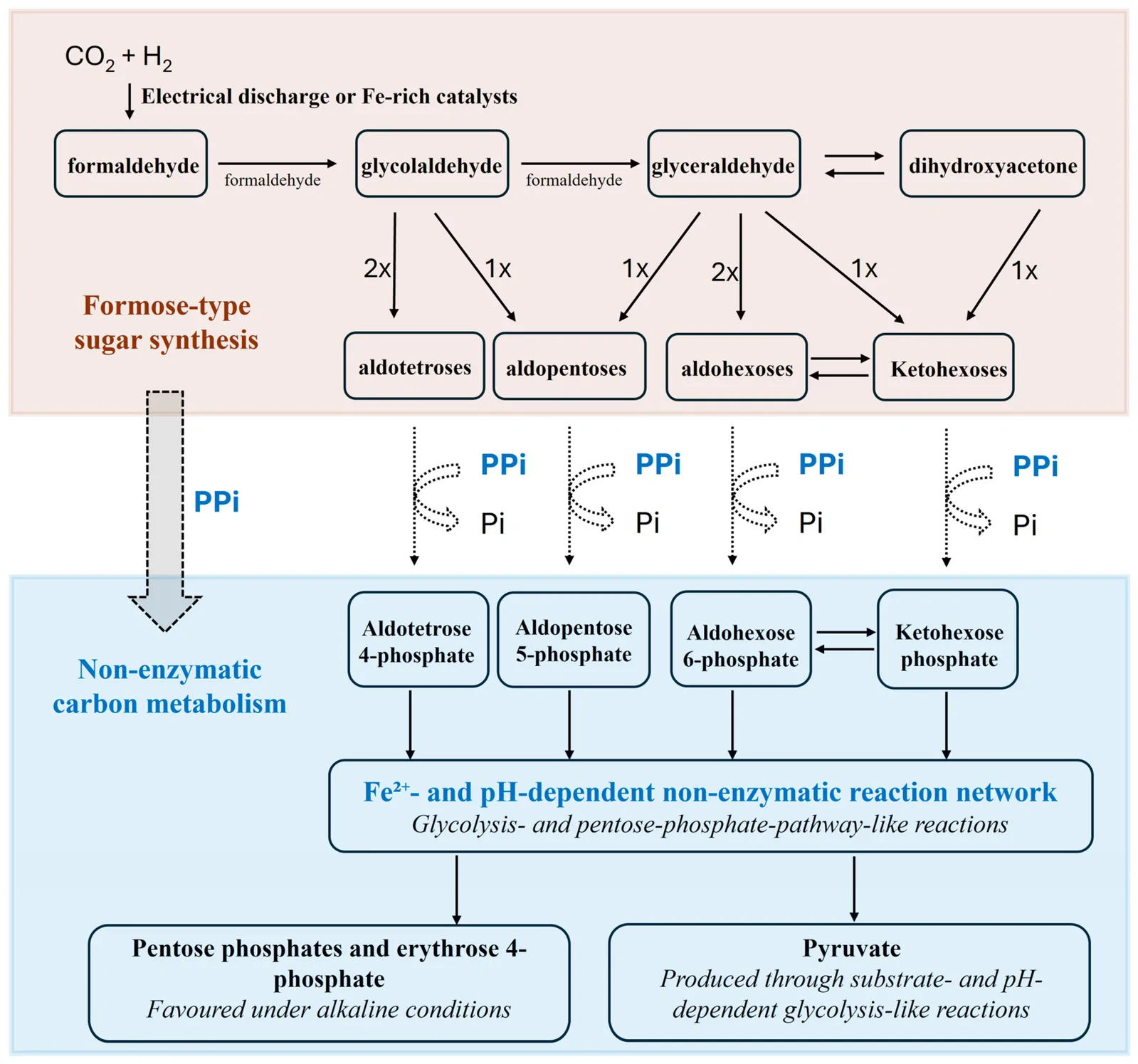
Proposed role of inorganic pyrophosphate in linking prebiotic sugar formation to primitive carbon metabolism. Formaldehyde generated from CO_2_ and H_2_ could enter formose-type chemistry and produce a heterogeneous pool of sugars. The model proposes that PP_i_ phosphorylated accessible terminal hydroxyl groups, generating sugar phosphates able to enter non-enzymatic reaction networks resembling glycolysis and the pentose phosphate pathway. Solid arrows indicate experimentally supported transformations; dashed arrows indicate the proposed PP_i_-dependent phosphorylation step.

Formaldehyde can be generated from CO_2_-containing, H_2_-rich atmospheres by electrical discharge [51,52] and from CO_2_ and H_2_ by catalysis with iron-rich meteoritic or volcanic particles at elevated temperatures and pressures [53,54] (Figure 3). Under alkaline, mineral-mediated conditions, formaldehyde can subsequently enter formose chemistry [55,56,57,58]. The initial formation of glycolaldehyde represents a major kinetic bottleneck in this reaction network [55,57], although recent experiments show that Mg-rich olivine can catalyse glycolaldehyde formation from formaldehyde in aqueous systems [56]. Once glycolaldehyde is available, aldol addition to formaldehyde can produce glyceraldehyde, followed by aldose–ketose isomerization and successive aldol and retro-aldol reactions that generate complex mixtures of tetroses, pentoses, and hexoses [55,57,58,59]. Minerals may additionally influence the stabilization or selection of particular products, including ribose [58,60].

### 3.2. Non-Enzymatic Transformations of Phosphorylated Carbon Intermediates

Once present, phosphorylated sugars could undergo further transformation without enzymes. Keller and colleagues showed that a broad panel of glycolytic and pentose-phosphate intermediates, including glucose 6-phosphate, fructose 6-phosphate, fructose 1,6-bisphosphate, dihydroxyacetone phosphate, glyceraldehyde 3-phosphate, 3-phosphoglycerate, 2-phosphoglycerate, phosphoenolpyruvate, and ribose 5-phosphate, underwent spontaneous interconversion in heated aqueous solutions, with Fe^2+^ promoting additional reactions and funneling carbon from several substrates towards pyruvate [61]. The structure of this non-enzymatic network depended strongly on pH: neutral or mildly acidic conditions favoured glycolysis-like reactions, whereas alkaline conditions increased the activity of pentose-phosphate-pathway-like transformations and parts of lower glycolysis [62]. Piedrafita and colleagues subsequently showed that amino acids could further modulate these reactions, with cysteine acting together with Fe^2+^ to accelerate the conversion of 6-phosphogluconate into pentose phosphates, particularly ribose 5-phosphate [63].

### 3.3. Prebiotic Routes to Phosphorylated Sugars

The availability of phosphorylated substrates is also supported by several experimental routes. Hirakawa et al. showed that glucose and gluconate can be generated from formaldehyde and glycolaldehyde under alkaline formose-like conditions. In separate experiments, they demonstrated that thermal evaporation of glucose or gluconate with orthophosphate, urea, and borate produced glucose 6-phosphate and 6-phosphogluconate, respectively, with phosphorylation occurring preferentially at the terminal C6 hydroxyl group [64].

A complementary route begins with the C_2_ precursor itself. Krishnamurthy et al. converted glycolaldehyde into glycolaldehyde phosphate in near-neutral aqueous solution using amidotriphosphate and Mg^2+^, obtaining nearly quantitative analytical conversion and a 76% isolated yield [65].

Once formed, glycolaldehyde phosphate remained capable of carbon-chain growth. Its aldomerization in strongly alkaline solution produced hexose 2,4,6-triphosphates and, in the presence of formaldehyde, pentose 2,4-bisphosphates, with ribose 2,4-bisphosphate among the principal products [66]. Related reactions were reproduced under milder conditions within the interlayers of mixed-valence layered hydroxide minerals, which concentrated glycolaldehyde phosphate from dilute solution and promoted the formation of tetrose 2,4-bisphosphates and hexose 2,4,6-triphosphates [67]. When glycolaldehyde phosphate was combined with glyceraldehyde 2-phosphate under near-neutral mineral-mediated conditions, pentose 2,4-bisphosphates formed in yields of up to 25%, with ribose 2,4-bisphosphate accounting for approximately 48% of the pentose fraction [68].

### 3.4. Pyrophosphate as a Proposed Link Between Sugar Formation and Carbon Metabolism

In this context, inorganic pyrophosphate may have provided an energetic link between prebiotic sugar synthesis and primitive carbon metabolism (Figure 3). Coupling pyrophosphate consumption to phosphorylation of these primary alcohols would therefore be modestly exergonic, with an estimated thermodynamic margin of approximately 2–6 kJ mol^−1^. This interpretation is supported by the ability of pyrophosphate to transfer phosphate to organic acceptors in extant pyrophosphate-dependent kinases [17,18,26] and by the experimental phosphorylation of uridine with pyrophosphate under prebiotically relevant drying conditions [16].

By contrast, phosphorylation at the anomeric carbon of aldoses would be close to thermoneutral or endergonic and would therefore require an additional thermodynamic bias [43]. Because many products of formose chemistry contain accessible terminal primary hydroxyl groups, recurrently generated pyrophosphate could, in principle, have converted part of this heterogeneous sugar pool into phosphorylated intermediates able to participate in non-enzymatic glycolysis- and pentose-phosphate-pathway-like networks. Direct phosphorylation of formose-derived sugars by pyrophosphate has not yet been demonstrated and constitutes a central unresolved transition in the proposed model. The thermodynamic relationship and known phosphoryl-transfer capacity of pyrophosphate nevertheless define experimentally plausible boundary conditions for testing this reaction.

## 4. Regeneration of Pyrophosphate and Partial Energetic Recycling

### 4.1. General Principle of Pyrophosphate Regeneration

If pyrophosphate contributed to sustained early energy coupling, it must have been recurrently supplied or regenerated rather than simply consumed. Beyond thermal condensation, volcanic chemistry, and wet–dry cycling, activated phosphate compounds offer a potential route. Their hydrolysis free energies (Table 2) are generally sufficient to offset the energetic cost of pyrophosphate formation from orthophosphate under the reference conditions [69,70,71]. If orthophosphate replaces water as the nucleophile, this energy can be retained in the phosphoanhydride bond of pyrophosphate rather than lost through hydrolysis.

### 4.2. Phosphoenolpyruvate

**Figure 4:**
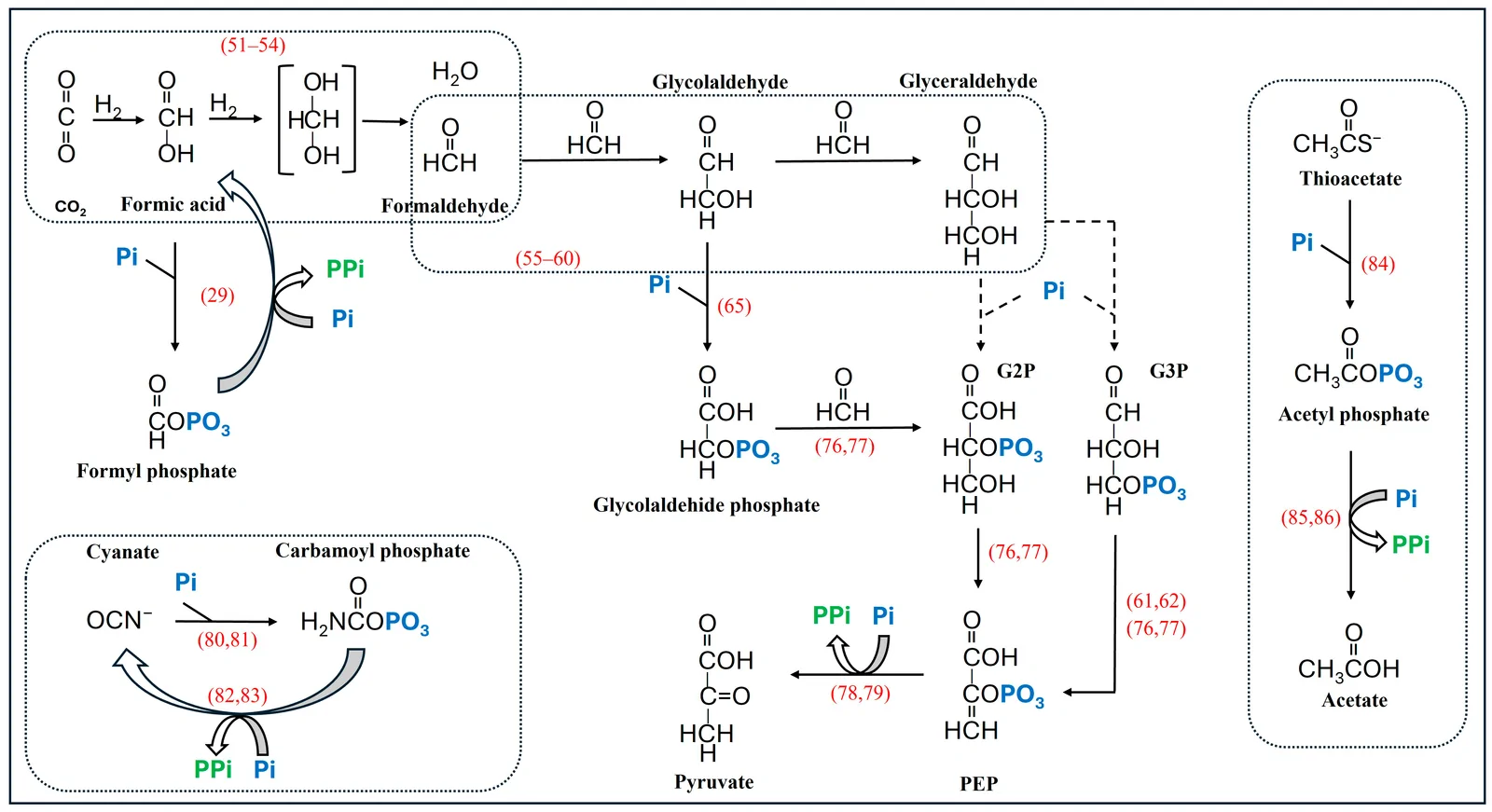
Experimentally supported routes to pyrophosphate regeneration from activated phosphate donors. Phosphoenolpyruvate, carbamoyl phosphate, and acetyl phosphate can transfer phosphoryl groups to orthophosphate (Pi), generating pyrophosphate (PPi) under mineral-assisted or low-water-activity conditions. Solid arrows indicate experimentally demonstrated transformations; dashed arrows indicate proposed or indirectly supported steps. PEP, phosphoenolpyruvate; G2P, glyceraldehyde 2-phosphate; G3P, glyceraldehyde 3-phosphate [29,51,52,53,54,55,56,57,58,59,60,61,62,65,76,77,78,79,80,81,82,83,84,85,86].

Phosphoenolpyruvate provides the clearest example (Figure 4). A plausible prebiotic route begins with glycolaldehyde phosphate, which can be formed by phosphorylation of glycolaldehyde with amidotriphosphate in near-neutral water [65]. It then reacts with formaldehyde to give glyceraldehyde 2-phosphate, which readily dehydrates to phosphoenol pyruvaldehyde [76]. Proline and orthophosphate promote the same C_2_ +  C_1_  sequence in neutral water, yielding phosphoenol pyruvaldehyde as the major product when formaldehyde is limiting [77]. Oxidation of the aldehyde affords phosphoenolpyruvate, with high yields reported using manganese dioxide and cyanide or chlorite and dimethyl sulfoxide, and lower yields with Fe^2+^ and hydrogen peroxide [76]. Once formed, phosphoenolpyruvate can transfer its phosphoryl group directly to orthophosphate: phosphoenolpyruvate + orthophosphate → pyruvate + pyrophosphate. Its large hydrolysis free energy gives this reaction a substantial formal thermodynamic margin (Table 2). Pyrophosphate formation has been observed on precipitated magnesium phosphate under reduced water activity, with approximately one-third of the adsorbed phosphoenolpyruvate converted after four days [78]. Related experiments with calcium-phosphate minerals also produced pyrophosphate, showing that mineral adsorption can divert enol-phosphate hydrolysis towards phosphoanhydride formation [79].

### 4.3. Carbamoyl Phosphate

Carbamoyl phosphate follows a shorter route (Figure 4). Cyanate reacts reversibly with orthophosphate in water to form carbamoyl phosphate [80]. Under mildly acidic to neutral conditions, cyanate-mediated phosphate activation produces carbamoyl phosphate as a transient intermediate, which can be trapped by imidazole; wet–dry cycling allows the reaction to proceed from millimolar starting concentrations by concentrating the reactants during evaporation [81]. Orthophosphate can also act as the acceptor in the subsequent reaction: carbamoyl phosphate + orthophosphate → cyanate + pyrophosphate. The energetic margin is favourable (Table 2), but mineral capture appears to be important. Pyrophosphate was detected when carbamoyl phosphate was incubated with precipitated calcium phosphate, with kinetics consistent with adsorption before phosphoryl transfer [82]. Cyanate-mediated pyrophosphate formation has also been demonstrated using brushite, further linking transient carbamoyl phosphate formation to mineral-assisted phosphate condensation [83]. These reactions define a continuous sequence in which cyanate first activates orthophosphate and the resulting carbamoyl phosphate transfers its phosphoryl group to a second phosphate molecule.

### 4.4. Acetyl Phosphate

Acetyl phosphate provides a direct connection between sulfur chemistry and pyrophosphate formation (Figure 4). Thioacetate reacts with orthophosphate in water to form acetyl phosphate within minutes at pH 6–8 and 20–50 °C, under both oxic and anoxic conditions [84]. Yields were modest, reaching approximately 2%, but the reaction required neither enzymes nor strongly dehydrating conditions. Mg^2+^ enhanced acetyl-phosphate formation in some experiments, whereas Ca^2+^ suppressed it by decreasing soluble phosphate. Methyl thioacetate was unreactive under the same conditions, indicating that free thioacetate is the more effective precursor. Acetyl phosphate can then undergo phosphorolysis: acetyl phosphate + orthophosphate → acetate + pyrophosphate. This reaction has been demonstrated in aqueous solution and is favoured by reduced water activity and divalent cations [85]. Calcium- and magnesium-phosphate precipitates further promote pyrophosphate formation through heterogeneous catalysis [86]. The sequence thioacetate → acetyl phosphate → pyrophosphate therefore provides an experimentally supported route by which the transfer potential of a sulfur-containing compound could be conserved in a phosphate–phosphate bond. The prebiotic availability of free thioacetate remains uncertain, because metal-sulfide experiments have primarily yielded thioesters such as methyl thioacetate rather than free thioacetic acid [87]. 

### 4.5. Additional Candidate Donors

Other activated donors remain plausible but less well supported. The acyl-phosphate group of 1,3-bisphosphoglycerate has sufficient transfer potential to drive pyrophosphate formation (Table 2), and an enzyme from *Neurospora crassa* can transfer its C_1_ phosphoryl group to phosphate [88]. This establishes that the bond can support phosphate–phosphate coupling, although direct transfer to free orthophosphate has not been shown.

Formyl phosphate is the simplest acyl-phosphate candidate. It is short-lived in neutral water, but its hydrolysis at pH 7 involves P–O bond cleavage, suggesting that the phosphorus centre may be accessible to attack by orthophosphate [89,90]. Direct formation of pyrophosphate from isolated formyl phosphate has not been demonstrated. However, heating ammonium formate with orthophosphate at pH 8 and 100 °C under drying conditions produced pyrophosphate in 53% yield, and transient formyl phosphate was proposed as an intermediate [29]. The intermediate was not detected, so the mechanism remains unresolved, but the experiment shows that formate-containing systems can promote substantial phosphate condensation under low-water-activity conditions.

Phosphocreatine is also thermodynamically capable of transferring a phosphoryl group to orthophosphate (Table 2), but no non-enzymatic formation of pyrophosphate from phosphocreatine has been reported. Its value is therefore mainly comparative: it shows that activated P–N bonds, as well as enol- and acyl-phosphate bonds, could in principle support phosphoanhydride synthesis. Simpler phosphoramidates are likely to be more relevant to prebiotic chemistry.

### 4.6. Experimental Status and Partial Energetic Closure

Phosphoenolpyruvate, carbamoyl phosphate, and acetyl phosphate currently have the strongest experimental support. Each can be produced through non-enzymatic chemistry and has been shown, in separate experimental systems, to support pyrophosphate formation. In each case, high local phosphate concentration, reduced water activity, or adsorption to phosphate-mineral phases favoured phosphate–phosphate coupling. The remaining donors broaden the chemical scope of the model but still require direct experimental testing.

These reactions do not yet constitute a reconstructed self-sustaining cycle. They instead support the more limited concept of partial energetic recycling: products generated within a carbon or sulfur reaction network may return activated phosphate to orthophosphate, thereby reducing—but not eliminating—the requirement for external pyrophosphate production.

## 5. What Modern Biology Demonstrates—And What It Does Not

**Figure 5:**
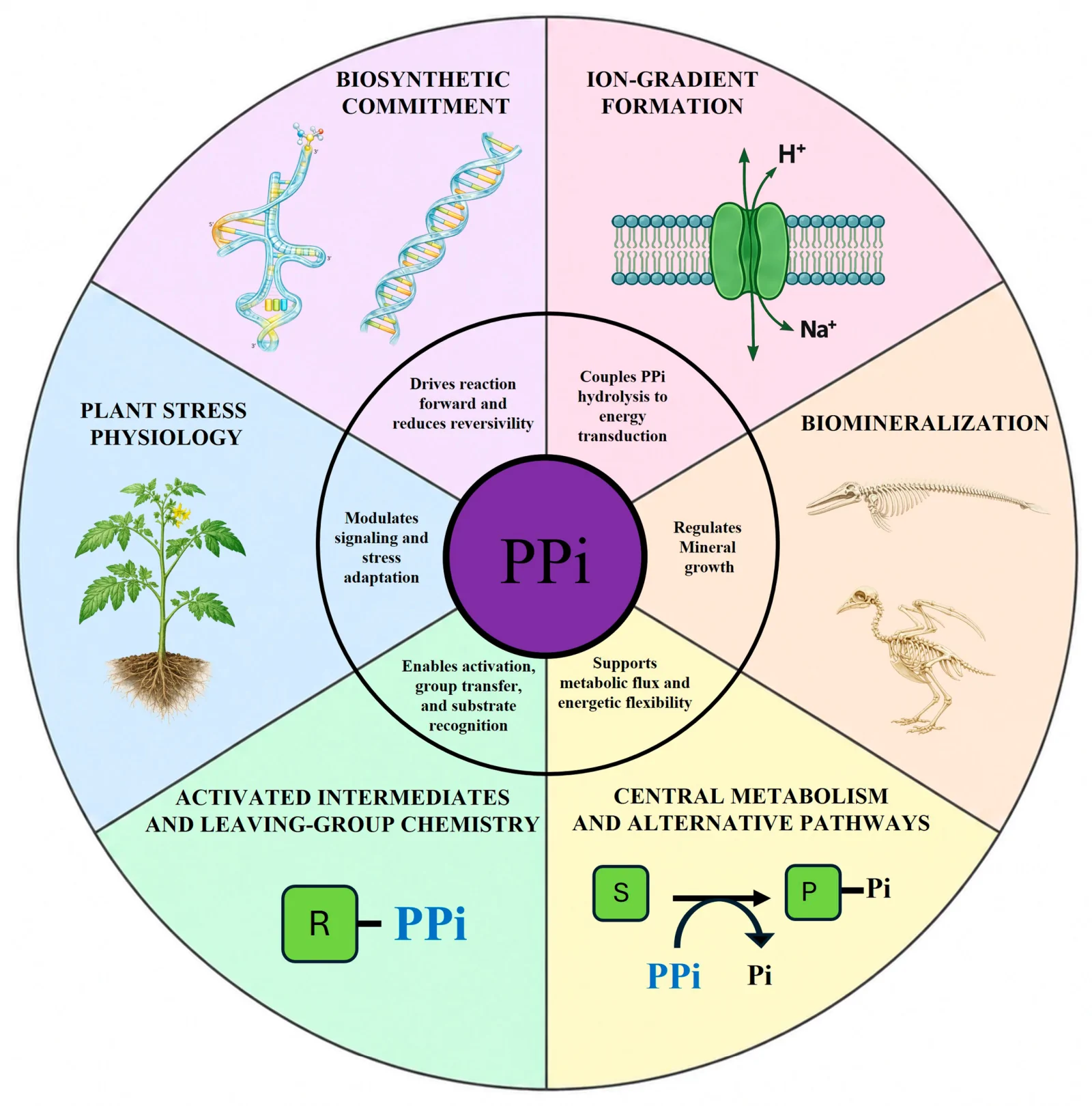
Roles of inorganic pyrophosphate in modern biology. Pyrophosphate (PP_i_) contributes to biosynthetic directionality, phosphoryl transfer, membrane energization, carbon metabolism, and mineralization control. Its function depends on context: PP_i_ may be produced, hydrolysed, transferred, or maintained to regulate reaction flux and physiological processes.

Modern metabolism is not a fossil record of prebiotic chemistry, and persistence of a reaction does not by itself establish antiquity. Modern systems can nevertheless demonstrate the chemical operations that pyrophosphate is capable of supporting. These include reinforcement of reaction directionality, reversible phosphoryl transfer, formation of activated metabolites, ion translocation, and regulation of mineral deposition (Figure 5).

### 5.1. Reaction Directionality and Biosynthetic Commitment

The most widespread of these functions is the reinforcement of biosynthetic commitment, used here in the conventional biochemical sense of reduced reversibility and increased forward reaction flux. A particularly common strategy is the cleavage of ATP to AMP and pyrophosphate during biosynthetic activation. In these reactions, ATP generates an activated intermediate while pyrophosphate is released as a leaving product (Table S1); its subsequent hydrolysis suppresses reversal and commits the intermediate to downstream synthesis [21,38]. In DNA polymerases, time-resolved crystallography has shown that this hydrolysis can occur within the polymerase active site after phosphodiester-bond formation, directly reducing the probability of pyrophosphorolysis [41]. In other pathways, the same effect is supplied by soluble or membrane-bound pyrophosphatases.

### 5.2. Diphosphate Groups in Substrate Activation and Molecular Recognition

A related use is found in activated metabolites. PRPP, nucleotide sugars, and isoprenoid diphosphates retain a diphosphate group as part of their molecular structure. Diphosphate groups are also built into several central cofactors, including thiamine pyrophosphate, NAD^+^, NADP^+^, FAD, and coenzyme A, where they contribute to molecular recognition, group transfer, or leaving-group chemistry. These compounds cannot be regarded as direct evidence for a prebiotic pyrophosphate system, but they show how deeply diphosphate chemistry became embedded in metabolic architecture. The diphosphate group is sufficiently stable for the metabolite to persist in solution, yet becomes an effective leaving group when correctly positioned by an enzyme and coordinated by metal ions. In PRPP, departure of pyrophosphate exposes the anomeric carbon of ribose to nucleophilic attack during nucleotide and amino acid biosynthesis [50]. Nucleotide sugars use nucleoside phosphates in an analogous way during glycosyl transfer, whereas ionization of prenyl diphosphates initiates many of the carbon–carbon bond-forming reactions of isoprenoid metabolism. Pyrophosphate chemistry therefore provides a balance between kinetic stability and latent reactivity [91].

The incorporation of diphosphate groups into cofactors is also reflected in protein structure. The Rossmann fold, one of the most widespread and evolutionarily ancient protein architectures, binds ribose-based cofactors such as NAD^+^, NADP^+^, and FAD through a conserved β–α–β framework in which a glycine-rich loop and the adjacent α-helix interact with the cofactor diphosphate group [92,93]. Comparative analyses suggest that major Rossmann-fold families descended from a common ancestor already capable of binding nucleotide-like cofactors before their diversification into distinct catalytic functions [94,95]. This does not imply that the fold evolved to recognize free inorganic pyrophosphate, but it provides a structural parallel to the biochemical persistence of diphosphate chemistry in metabolism.

### 5.3. Direct Conversion into Electrochemical Work

Membrane pyrophosphatases use the same bond energy for a different purpose (Figure 5). These pumps couple pyrophosphate hydrolysis to the movement of H^+^, Na^+^, or both ion across a membrane in some enzymes. The resulting gradient supports pH regulation, ion homeostasis, and secondary transport in prokaryotes, protists, and plants [20,36,37,96]. Here, pyrophosphate is not simply removed to make another reaction irreversible; its hydrolysis is converted directly into electrochemical work.

### 5.4. Reversible Phosphoryl Transfer and Carbon Flux

Pyrophosphate also remains linked to carbon flux. As discussed above, pyrophosphate-dependent phosphofructokinase uses it to phosphorylate fructose 6-phosphate without consuming ATP. Pyruvate phosphate dikinase extends this chemistry to the interconversion of pyruvate and phosphoenolpyruvate. The enzyme catalyses the reversible reaction ATP + pyruvate + phosphate ⇌ AMP + phosphoenolpyruvate + pyrophosphate. In C_4_ plants, the reaction normally proceeds towards phosphoenolpyruvate formation and releases pyrophosphate; in several microorganisms, the reverse direction can consume pyrophosphate and AMP while forming ATP. The reaction is mediated through a phosphorylated histidine that transfers phosphoryl groups between spatially separated active sites. Pyrophosphate is therefore linked not only to carbon conversion, but also to the balance between ATP, AMP, and phosphoenolpyruvate [97,98].

### 5.5. Context-Dependent Regulatory Functions

Outside the cytosol, pyrophosphate has a largely regulatory role in biomineralization. Extracellular pyrophosphate binds to nascent calcium-phosphate crystals and restrains hydroxyapatite growth [99,100]. Its concentration is set by opposing activities: ectonucleotide pyrophosphatase/phosphodiesterase 1 generates extracellular pyrophosphate from nucleotides, whereas tissue-nonspecific alkaline phosphatase hydrolyses pyrophosphate to phosphate [101]. Mineral deposition therefore depends partly on the local pyrophosphate/phosphate balance [102]. Excess pyrophosphate impairs skeletal and dental mineralization, whereas insufficient pyrophosphate favours ectopic calcification in soft tissues and blood vessels [103]. In this setting, pyrophosphate functions as a boundary condition for crystal growth rather than as an energy source.

Plant physiology brings several of these functions together. The vacuolar H^+^-pyrophosphatase AVP1 consumes cytosolic pyrophosphate while contributing to the proton gradient across the tonoplast [104]. Increased AVP1 expression has been associated with improved growth under drought and salinity, initially attributed to stronger vacuolar ion sequestration [105]. Later work showed that control of cytosolic pyrophosphate is equally important. Arabidopsis plants lacking functional AVP1 accumulate pyrophosphate, which inhibits gluconeogenesis and restricts postgerminative growth; expression of a soluble cytosolic pyrophosphatase rescues this phenotype without restoring proton pumping [106]. AVP1 therefore sits at the intersection of ion transport, carbon metabolism, and pyrophosphate homeostasis, which probably accounts for its broad effects on growth and stress adaptation [104].

### 5.6. Limits of Evolutionary Inference

These roles make pyrophosphate difficult to classify as either an energy donor or a metabolic by-product. Its function depends on context. In biosynthesis, its removal commits reactions to product formation; in activated metabolites, its departure enables group transfer; in membrane pumps, its hydrolysis performs electrochemical work; and in extracellular fluids, its persistence limits mineral growth. Its broad distribution does not demonstrate that pyrophosphate had the same functions before the emergence of modern metabolism. It does, however, show that the same chemical linkage has been repeatedly retained to control directionality, activation, molecular recognition, and energy transfer.

## 6. Activated Pentose Phosphates as Candidate Transitional Molecular Carriers

### 6.1. Why Pyrophosphate Alone Is Insufficient

Modern biochemistry uses ATP to generate activated intermediates for the synthesis of nucleic acids, peptides, lipids, and cofactors. These reactions commonly exploit cleavage of ATP at the AMP–diphosphate bond, either through adenylyl transfer with the release of pyrophosphate (Table S1) or through direct transfer of the β,γ-diphosphoryl group, as in PRPP synthesis). Both strategies provide substantial thermodynamic leverage, with an associated standard transformed free-energy change of approximately −46 kJ mol^−1^ [34,38]. This energetic requirement presents a difficulty for a system based exclusively on pyrophosphate. Although pyrophosphate hydrolysis could support the formation of relatively low-energy phosphate monoesters, it would not by itself provide sufficient thermodynamic driving force to generate phosphoanhydride bonds equivalent to those in ADP or ATP. Pyrophosphate could therefore have contributed to the initial phosphorylation of a ribose scaffold, but would not readily convert a ribose phosphate or nucleotide monophosphate into the corresponding di- or triphosphorylated compound. The capacity of activated phosphate donors (Table 2) to support phosphoanhydride synthesis is supported by direct chemical and biochemical precedents. Acetyl phosphate can phosphorylate ADP to ATP non-enzymatically in water [84]. This reaction was subsequently confirmed at yields of approximately 15–20% in mildly acidic aqueous solution in the presence of Fe^3+^. Under the same conditions, carbamoyl phosphate also supported ATP formation, although less efficiently [8]. Pyruvate phosphate dikinase provides a complementary biochemical example, coupling the conversion of PEP to pyruvate with ATP formation from AMP and pyrophosphate [98]. The same principle is illustrated by enzyme-catalysed ATP formation from ADP using PEP [107], 1,3-bisphosphoglycerate [108], carbamoyl phosphate [109], phosphocreatine [110], or acetyl phosphate [111]. Although these reactions do not establish a prebiotic pathway, they demonstrate that such energetic couplings are biochemically feasible.

### 6.2. Chemical Accessibility and Recognition of Pentose Polyphosphates

**Figure 6:**
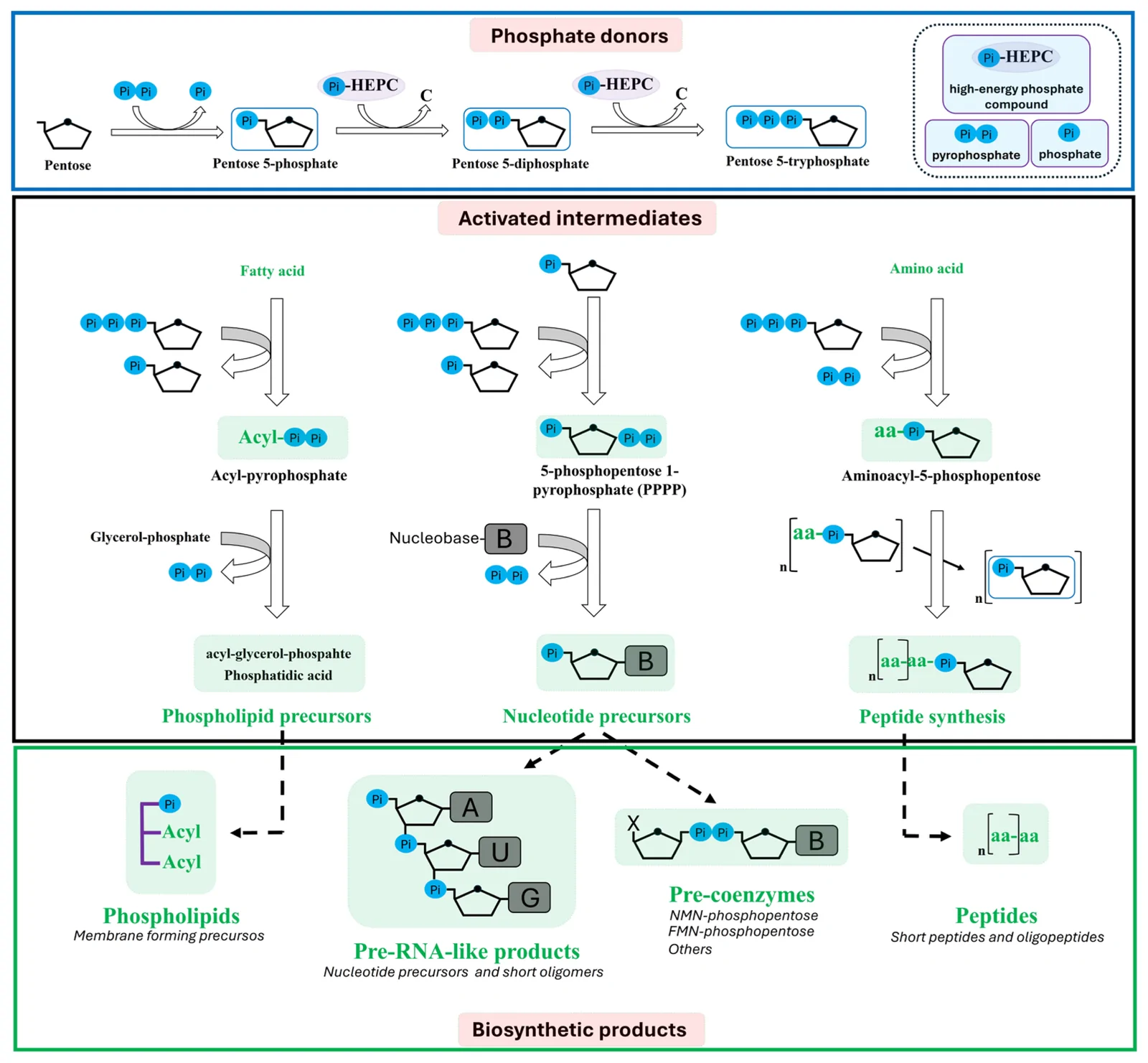
Hypothetical transition from pyrophosphate-based chemistry to nucleotide-dependent biosynthesis. Activated phosphate donors could have supported pyrophosphate regeneration and the formation of pentose diphosphates and triphosphates. These compounds are proposed as transitional carriers for phosphoribosyl, acyl, and aminoacyl transfer, linking phosphate-energy metabolism to nucleotide, lipid, and peptide synthesis. Dashed lines indicate hypothetical transitions, and the crossed reaction denotes a thermodynamically or chemically unfavourable step. HEPC, high-energy phosphate compound; Pi, inorganic phosphate; PPi, pyrophosphate; PEP, phosphoenolpyruvate.

Moreover, a pentose phosphate could, in principle, accept additional phosphoryl groups from the same activated donors (Figure 6). Although these phosphorylation reactions have not been demonstrated with the compounds listed in Table 2, ribose 5-diphosphate and ribose 5-triphosphate have been synthesized and characterized [112]. Polyphosphorylated pentoses are also recognized by modern enzymes: ribose-5-phosphate isomerase acts on ribose 5-diphosphate and ribose 5-triphosphate [113], whereas ribose 5-triphosphate competitively inhibits *Escherichia coli* RNA polymerase [114], indicating recognition of the ribose–polyphosphate moiety. Here, pentose diphosphates and triphosphates refer to compounds containing P–O–P chains attached to the pentose scaffold, not to pentoses bearing independent phosphate monoesters at multiple carbon positions.

### 6.3. Phosphoribosyl Transfer, Nucleotides, and Cofactors

#### 6.3.1. PRPP-like Chemistry and Nucleotide Formation

In the model shown in Figure 6, transfer of a diphosphoryl group to the anomeric hydroxyl of pentose 5-phosphate would generate a PRPP-like intermediate. In modern metabolism, PRPP is formed by transfer of the β,γ-diphosphoryl group of ATP to ribose 5-phosphate, releasing AMP, and subsequently transfers its phosphoribosyl moiety to nitrogenous compounds with the release of pyrophosphate [50]. A direct prebiotic precedent is provided by the non-enzymatic formation of PRPP from ribose and inorganic phosphate on fumed silica, where it acted as an intermediate in the one-pot synthesis of AMP from adenine, ribose, and phosphate [115]. These results demonstrate that both PRPP formation and phosphoribosyl transfer to a nucleobase are chemically feasible, although production of the proposed intermediate from pentose triphosphate remains to be demonstrated.

#### 6.3.2. Incorporation into Nucleotide-Derived Cofactors

A PRPP-like intermediate could have connected phosphate-based energy metabolism with the synthesis of nucleotides and nucleotide-derived cofactors. Reaction with preformed purine or pyrimidine bases would resemble modern salvage pathways, whereas the resulting nucleotides could subsequently be phosphorylated and incorporated into RNA. PRPP also participates directly in modern NAD biosynthesis [50], and prebiotic experiments have demonstrated the formation of a phosphorylated nicotinamide riboside from an activated ribose phosphate [116]. More generally, the occurrence of nucleotide moieties in NAD, FAD, coenzyme A, and related cofactors is consistent with an early role for ribonucleotide derivatives as carriers of chemically reactive groups [117]. Formation of the complete cofactors, however, would have required additional activation and coupling reactions.

The heterocyclic components required for this transition were also chemically accessible. The nicotinamide moiety is likewise accessible through non-enzymatic chemistry, and both phosphorylated nicotinamide riboside and complete NAD^+^ have been generated under proposed prebiotic conditions [116,118]. Evidence for flavin synthesis is less complete: a key condensation leading to the riboflavin precursor 6,7-dimethyl-8-ribityllumazine proceeds spontaneously in dilute aqueous solution at neutral pH, but a continuous prebiotic synthesis of riboflavin or FAD from simple precursors has not yet been demonstrated [119].

#### 6.3.3. Availability and Specialization of Nucleobases

The canonical nucleobases were unlikely to have been equally available under prebiotic conditions. Adenine has been produced repeatedly in diverse chemical settings. In the classical experiments of Oró and Kimball, heating concentrated HCN in aqueous ammonia at moderate temperatures generated substantial amounts of adenine, and Oró subsequently proposed plausible pathways involving HCN oligomers and imidazole intermediates [120,121]. Adenine formation has also been demonstrated in dilute frozen HCN solutions and from the HCN tetramer diaminomaleonitrile in the presence of ammonium formate [122,123]. Pyrimidines were likewise chemically accessible: concentrated urea reacts with cyanoacetaldehyde to produce cytosine and uracil [124]. Cytosine, however, is comparatively unstable and undergoes spontaneous hydrolytic deamination to uracil, making uracil a potentially more persistent prebiotic substrate [125]. Adenine and uracil may therefore have been among the most readily available substrates for early phosphoribosyl transfer.

Their incorporation into pentose-phosphate carriers could have favoured the replacement of simpler phosphorylated pentoses by nucleotides with additional recognition and catalytic properties. Subsequent functional specialization may then have selected adenine nucleotides for phosphoryl and acyl activation, ultimately giving rise to AMP- and ATP-based chemistry, whereas uridine nucleotides became central to glycosyl transfer. In modern metabolism, UTP reacts with sugar 1-phosphates to generate UDP-sugars with the release of pyrophosphate, and UDP-glucose provides the activated glucosyl donor used in the polymerization of glucose into glycogen and cellulose [126,127,128]. Cytidine nucleotides underwent a complementary specialization in membrane biosynthesis. CTP reacts with phosphatidic acid to form CDP-diacylglycerol with the release of pyrophosphate; this activated lipid subsequently donates its phosphatidyl group during the synthesis of phosphatidylglycerol, cardiolipin, and phosphatidylinositol [129,130]. CTP also activates phosphocholine and phosphoethanolamine as CDP-choline and CDP-ethanolamine, which transfer their phosphobase moieties to diacylglycerol during the synthesis of phosphatidylcholine and phosphatidylethanolamine [131].

### 6.4. Possible Acyl-Transfer Functions in Primitive Lipid Synthesis

In the proposed model shown in Figure 6, a pentose triphosphate would activate a fatty acid, releasing pentose 5-phosphate and generating an acyl-pyrophosphate donor. Transfer of the acyl group to glycerol phosphate would produce a lysophosphatidic-acid-like intermediate, and a second acylation would yield phosphatidic acid. A direct biochemical precedent is provided by the bacterial PlsX–PlsY pathway: PlsX forms acyl phosphate from acyl-ACP, and PlsY transfers the acyl group directly to glycerol 3-phosphate to generate lysophosphatidic acid [132,133]. The second acyl chain is introduced in modern cells by PlsC using acyl-ACP, so the complete two-step acyl-phosphate route proposed here remains hypothetical.

Prebiotic experiments nevertheless support the underlying chemistry. Activated fatty acids acylate glycerol phosphate non-enzymatically, producing mono- and diacylglycerol phosphates; medium-chain products subsequently form vesicles capable of retaining nucleotides [134]. These results establish the feasibility of fatty-acid activation, glycerol-phosphate acylation, and membrane self-assembly, although the proposed pentose-phosphate carrier has not been tested.

Moreover, fatty acids could have been supplied by several abiotic processes. Fischer–Tropsch-type reactions under hydrothermal conditions produce mixtures of straight-chain fatty acids, alcohols, and hydrocarbons spanning biologically relevant chain lengths [135,136]. Mixed medium-chain amphiphiles generated by this chemistry can also assemble into vesicles under saline and alkaline conditions [137]. Glycerol phosphate was likewise chemically accessible: heating glycerol with orthophosphate in the presence of urea or cyanamide produced glycerophosphates, predominantly *sn*-glycerol 1(3)-phosphate [138]. Its formation could also, in principle, be driven by pyrophosphate-dependent phosphorylation. Hydrolysis of *sn*-glycerol 3-phosphate releases only about 9 kJ mol^−1^ [139]. Its formation from glycerol could therefore, in principle, be driven by pyrophosphate hydrolysis, although direct pyrophosphate-dependent phosphorylation of glycerol has not been demonstrated.

### 6.5. Possible Aminoacyl-Transfer Functions in Primitive Peptide Synthesis

In the model shown in Figure 6, pentose triphosphate would activate the carboxyl group of an amino acid by transferring its pentose-phosphate moiety, generating an aminoacyl–pentose phosphate intermediate and releasing pyrophosphate, analogous to the formation of aminoacyl-AMP from ATP in modern metabolism [39,140]. Nucleophilic attack by the amino group of a second aminoacyl–pentose phosphate intermediate would then form a peptide bond while retaining an activated terminal carboxyl group, thereby allowing repeated cycles of chain extension. The proposed activation step has not been demonstrated with pentose triphosphate, but its underlying chemistry has strong experimental precedents. Aminoacyl phosphates, which are simplified analogues of biological aminoacyl adenylates, react with amino groups in water to form dipeptides and longer oligomers [141,142]. Trimetaphosphate likewise promotes peptide formation over a broad range of aqueous conditions [143,144]. Moreover, preformed aminoacyl phosphate–oligonucleotide conjugates support template-directed peptide synthesis, showing that an activated aminoacyl linkage attached to a sugar–phosphate scaffold can combine acyl transfer with molecular recognition [145]. These observations support the feasibility of phosphate-mediated peptide synthesis, although formation of the specific pentose-derived intermediate proposed here remains to be demonstrated.

## 7. An Evolutionary Model: From Geochemical Pyrophosphate to ATP-Centred Bioenergetics

The evidence considered above supports a staged model in which inorganic pyrophosphate acted not as a fully developed universal energy currency, but as a transitional coupling intermediate between geochemical phosphate activation and nucleotide-based bioenergetics. Such a role is consistent with early proposals that pyrophosphate preceded ATP in biological energy conversion and with the capacity of pyrophosphate hydrolysis to support membrane energization and ATP synthesis [146,147]. In this framework, ATP did not emerge by abruptly replacing an unrelated prebiotic energy system. Rather, the adenylate scaffold consolidated functions previously distributed among pyrophosphate, enol and acyl phosphates, and phosphorylated carbon intermediates, while introducing the molecular recognition and catalytic selectivity required for increasingly regulated metabolism. The transition was therefore likely gradual, with pyrophosphate-dependent, substrate-level, and nucleotide-dependent modes of energy coupling coexisting before chemiosmotic ATP regeneration became dominant [148]. The prebiotic formation of phosphoribosyl pyrophosphate and AMP on mineral surfaces provides a possible chemical bridge between activated phosphate chemistry and nucleotide-based activation [115]. The stages outlined below should therefore be understood as functionally separable transitions within this model rather than as a uniquely ordered historical sequence; some may have overlapped, occurred in parallel, or been connected through alternative chemical routes.

### 7.1. Stage I: Local Geochemical Production of Pyrophosphate

The first stage would have preceded metabolism and biological catalysis. Orthophosphate could have been converted into condensed phosphate species, including pyrophosphate in some systems, within spatially restricted environments in which heat, dehydration, or phosphorus redox chemistry promoted phosphate activation [14,27,28,30,31]. These processes need not have established a persistent global reservoir. Volcanic and geochemically reactive settings could instead have generated localized supplies of activated phosphate species, while evaporating pools and phosphate-rich mineral interfaces could have concentrated them and prolonged their contact with potential organic acceptors (Figure 1). Such short-lived inventories could have been chemically effective if local production or concentration outpaced hydrolysis long enough for pyrophosphate to participate in phosphate-transfer reactions. Wet–dry cycling would have been particularly favourable because evaporation concentrates solutes and lowers water activity, whereas limited rehydration restores a liquid phase in which reactants and products can become mobile [32].

**Figure 7:**
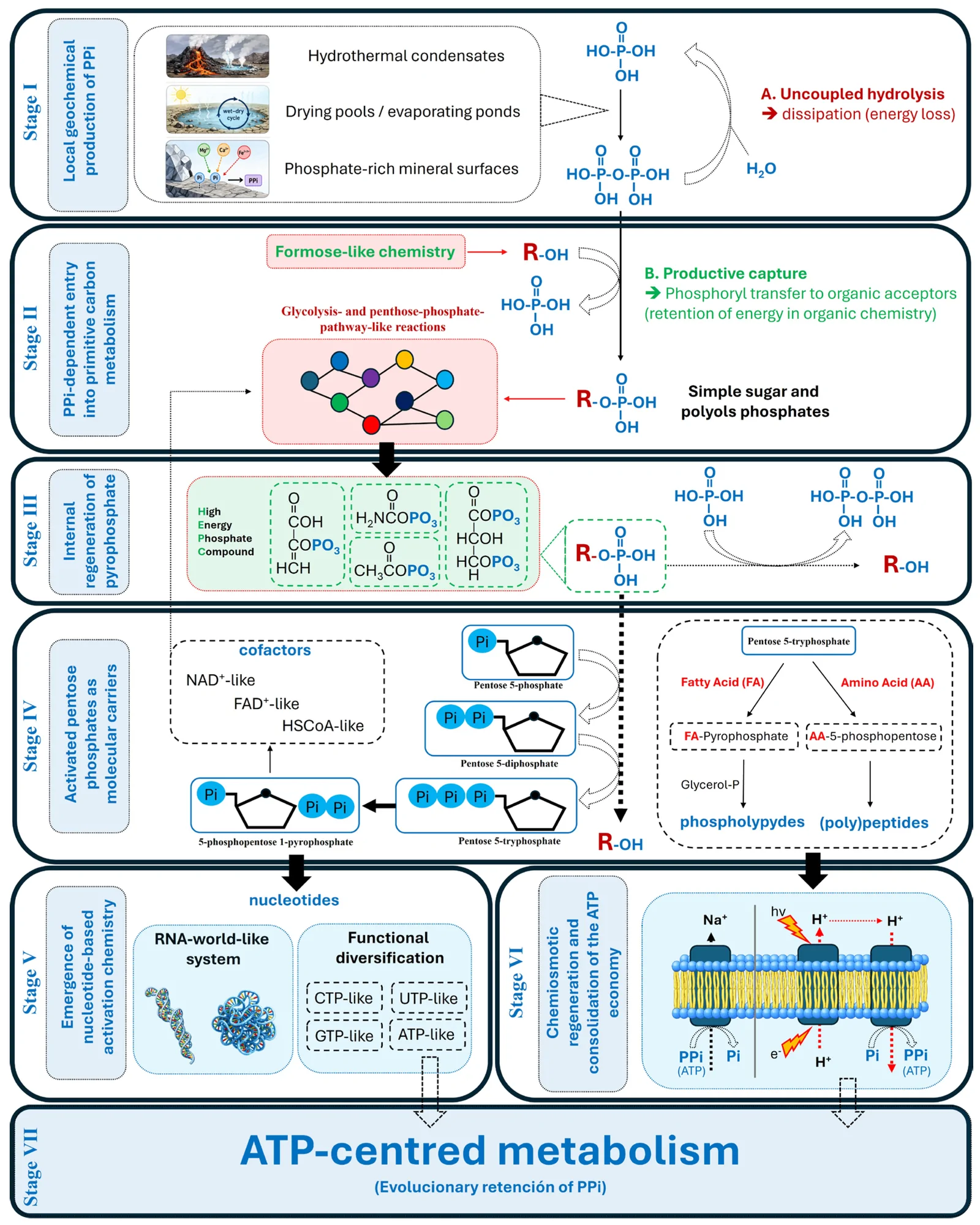
Proposed transition from geochemical pyrophosphate chemistry to ATP-centred metabolism. The model begins with recurrent local production of pyrophosphate and its productive capture through phosphorylation of simple carbon compounds. Phosphorylated carbon networks could then generate activated phosphate donors able to regenerate pyrophosphate and support the formation of activated pentose phosphates. These compounds are proposed as transitional carriers connecting phosphate-energy metabolism with nucleotide, cofactor, lipid, and peptide synthesis. The emergence of nucleotide-based activation chemistry and chemiosmotic ATP regeneration would ultimately have favoured an ATP-centred metabolic economy, while retaining pyrophosphate in specialized functions. The stages are functionally distinct and may have overlapped or proceeded in parallel. Pi, inorganic phosphate; PPi, pyrophosphate.

In such settings, pyrophosphate could have coupled episodic environmental disequilibria to organic chemistry. Formation of its phosphoanhydride bond would have retained part of the free energy supplied by heating, dehydration, or redox conversion. Uncoupled hydrolysis would have dissipated this energy, whereas phosphoryl transfer to an organic acceptor could have retained part of it in a more persistent organophosphate product (Figure 7). The pyrophosphate-dependent phosphorylation of uridine under drying conditions provides experimental proof of principle that such transfer can occur without enzymes, with the highest product yields obtained when reduced water activity, quartz, Mg^2+^, and urea were combined [16]. The defining feature of this stage was therefore not the long-term stability of pyrophosphate, but its recurrent local production or concentration and the productive use of its phosphoryl-transfer potential before hydrolytic loss.

### 7.2. Stage II: Capture of Pyrophosphate in Primitive Carbon Metabolism

A second stage would have emerged when pyrophosphate turnover became productively coupled to the phosphorylation of organic substrates (Figure 7). This chemistry was unlikely to have acted uniformly across the heterogeneous products of prebiotic carbon synthesis. Exposed primary hydroxyl groups on small polyols and sugars would have provided the most accessible acceptors, favouring the formation of terminal phosphate monoesters over more demanding modes of carbohydrate activation. The model does not require efficient or indiscriminate phosphorylation: a low flux could have been consequential if selected products persisted or were consumed by subsequent reactions. Direct phosphorylation of formose-derived sugars by pyrophosphate remains to be demonstrated, but enzyme-free phosphoryl transfer from pyrophosphate and preferential terminal phosphorylation through other prebiotic routes establish the chemical plausibility of this transition [16,64].

Phosphorylation would not, by itself, have resolved the compositional complexity of formose chemistry. Selection could instead have emerged from the coupling of phosphate transfer to differential mineral adsorption, metal coordination, local retention, or rapid downstream consumption. Under these conditions, a subset of phosphorylated products could have been channelled into non-enzymatic interconversion networks whose topology overlaps with glycolysis and the pentose phosphate pathway [61,62]. Pyrophosphate would thus have provided more than a source of activated phosphate: it could have linked geochemical energy input to a reaction-connected pool of carbon intermediates.

This stage would still have remained environmentally dependent. Pyrophosphate production was supplied externally, and chemical selectivity arose from the local physicochemical setting rather than from evolved catalysts. Its significance lies therefore not in the establishment of an autonomous metabolism, but in the first productive capture of geochemically generated phosphoanhydride energy within an organized carbon reaction network.

### 7.3. Stage III: Internal Regeneration and Partial Energetic Recycling

A critical step towards partial energetic autonomy would have occurred when the reaction network began to regenerate pyrophosphate from activated products formed within its own carbon and sulfur chemistry (Figure 7). Phosphoenolpyruvate provides the most direct connection to primitive carbon metabolism: plausible prebiotic reaction sequences can generate this enol phosphate, which can subsequently transfer its phosphoryl group to orthophosphate on precipitated phosphate minerals [76,78]. Carbamoyl phosphate offers a parallel route arising from cyanate chemistry, whereas acetyl phosphate could connect thioester formation to phosphate activation [82,84]. Acetyl phosphate phosphorolysis has been observed in aqueous solution and is strongly promoted by reduced water activity, divalent cations, and heterogeneous phosphate phases [85,86].

This chemistry defines a potential feedback loop: environmental pyrophosphate → phosphorylated carbon intermediates → activated phosphate donors → regenerated pyrophosphate.

The scheme should not be interpreted as an experimentally reconstructed closed cycle. Rather, its individual steps provide a plausible route to partial energetic closure. Once products formed within the network could return phosphoryl groups to orthophosphate, environmental pyrophosphate production would still initiate or replenish the system but would no longer be required for every turnover. Experiments in which orthophosphate is repeatedly activated and transferred during wet–dry cycling support the broader principle that phosphate activation can be sustained through recurrent physicochemical processing rather than a single activation event [81].

Before the emergence of replication, any resulting selection would have been chemical rather than Darwinian. Networks in which pyrophosphate regeneration approached or exceeded its loss through hydrolysis would have retained activated phosphate and organophosphate intermediates for longer periods. Such feedback could have increased network persistence, biased reaction flux towards productive pathways, and reduced dependence on the timing of external activation events. The evolutionary significance of this stage therefore lies not in complete metabolic autonomy, but in the first internal recycling of a coupling intermediate.

Such pre-replicative persistence should not be conflated with biological evolution. In the absence of a heritable information-bearing system, favourable chemical states could persist through recurrent environmental conditions or chemical feedback, but they could not undergo Darwinian cumulative adaptation. The present stage should therefore be understood as a pre-hereditary chemical transition rather than as an explanation for the origin of genetic information or biological evolution.

### 7.4. Stage IV: Incorporation of Activated Pentose Scaffolds as Candidate Molecular Carriers

Primitive carbon networks would also have supplied pentose monophosphates, introducing a stereochemically defined organic scaffold into phosphate-based energy coupling. The relevant transition, however, was not simply the addition of phosphate monoesters at multiple positions on the sugar (Figure 7). It required the formation of pentose diphosphates and triphosphates containing P–O–P phosphoanhydride bonds, such as ribose 5-diphosphate or ribose 5-triphosphate. These compounds should therefore be distinguished from pentoses carrying several independent phosphate monoesters. Although pyrophosphate could plausibly have contributed to the initial formation of pentose monophosphates, extension of a phosphate monoester into a diphosphate or triphosphate chain would have required a stronger source of activation. Enol and acyl phosphates generated within the emerging carbon network provide candidate donors for this step. Ribose 5-diphosphate and ribose 5-triphosphate have been synthesized, establishing the chemical accessibility of these structures, although not their formation under prebiotic conditions [112].

An activated pentose polyphosphate would have combined two properties that had previously resided in separate molecular classes: the transfer potential of a phosphoanhydride chain and the stereochemical information encoded by a chiral sugar scaffold. Recognition of ribose 5-triphosphate and related pentose diphosphates and triphosphates by RNA polymerase shows that the ribose–polyphosphate moiety can occupy molecular binding sites even in the absence of a nucleobase [114]. This observation does not imply an ancestral role for these compounds, but it illustrates how attachment of an activated phosphate chain to a pentose could have introduced a first level of substrate discrimination into phosphate-transfer chemistry.

The most direct function of such a carrier would have been phosphoribosyl transfer. Phosphoribosyl pyrophosphate (PRPP) provides the extant biochemical precedent: its activated anomeric centre transfers a phosphoribosyl group to nucleobases and other acceptors. The formation of PRPP and AMP from ribose, orthophosphate, and adenine on silica establishes that activated ribose phosphates can connect mineral-mediated phosphate chemistry to nucleotide synthesis [115]. More extensive roles in phosphoryl, aminoacyl, or acyl-group transfer remain possible, but should be presented as hypotheses rather than established properties of pentose diphosphates and triphosphates. Independent experiments show that aminoacyl phosphates can promote peptide-bond formation in water and that activated acyl chemistry can generate membrane-forming acylglycerol phosphates, demonstrating the accessibility of the downstream reaction classes envisaged here without establishing pentose diphosphates and triphosphates as their common carrier [134,141].

This remains the least constrained stage of the model. Existing studies establish that activated pentose diphosphates and triphosphates can be synthesized, that their sugar–polyphosphate framework can be recognized by modern proteins, and that PRPP-like chemistry can mediate phosphoribosyl transfer. They do not yet demonstrate the proposed evolutionary sequence: phosphorylation of pentose monophosphates by prebiotically available enol or acyl phosphates, persistence of the resulting products against hydrolysis, and their subsequent participation in enzyme-free group-transfer reactions. Experimental reconstruction of these steps will be required before activated pentose phosphates can be assigned to a credible intermediary position between simple phosphate donors and nucleotide-based carriers.

### 7.5. Stage V: Emergence of Nucleotide-Based Activation Chemistry

Activated pentose phosphates would have acquired a qualitatively new role once coupled to nitrogenous bases. Addition of a nucleobase converted a sugar–phosphate carrier into a nucleotide, introducing a recognition surface capable of selective molecular interactions, base pairing, and ultimately participation in catalysis (Figure 7). PRPP-like chemistry provides a concrete bridge for this transition. Mineral-mediated experiments have demonstrated the formation of phosphoribosyl pyrophosphate and AMP from ribose, orthophosphate, and adenine on silica, establishing that phosphoribosyl transfer can connect phosphate activation directly to nucleotide synthesis without enzymes [115]. Subsequent phosphorylation of the resulting nucleotides could have produced activated monomers for RNA-like polymers or furnished nucleotide components for primitive cofactors. Prebiotic routes to phosphorylated nicotinamide riboside further support a role for nucleotide scaffolds in group-transfer and redox chemistry, although the assembly of complete cofactors would have required additional activation steps [116,117].

Adenine nucleotides may have become preferential components of emerging activation chemistry because adenine can arise through several experimentally supported HCN-based routes [120,121,123]. Availability alone, however, cannot explain the later predominance of adenylates. Their advantage would have emerged from the combination of a recognizable molecular scaffold with a chemically versatile phosphate chain. As catalysts became more selective, adenylates could support phosphoryl and diphosphoryl transfer, adenylylation, and the formation of activated acyl intermediates. The division of labour evident in extant metabolism—adenylates in energy coupling and substrate activation, uridylates in glycosyl transfer, and cytidylates in membrane synthesis—may represent the endpoint of this progressive functional differentiation rather than evidence that each specialization arose simultaneously [126,133].

ATP would therefore have offered more than a larger thermodynamic driving force. Its adenosine moiety provided a binding handle for increasingly selective catalysts, whereas the triphosphate chain permitted chemically distinct cleavage and transfer reactions. Catalyst evolution could consequently direct the same molecular carrier towards different substrates and products while preserving a common mechanism of activation. In this sense, ATP was not simply a stronger alternative to pyrophosphate. It was a more addressable coupling molecule whose reactivity could be programmed by molecular recognition [8].

This stage remains a proposed evolutionary transition rather than a reconstructed pathway. Mineral-mediated PRPP and AMP formation, prebiotic nucleobase synthesis, and non-enzymatic production of nucleotide-derived cofactors establish several of its constituent steps, but they do not yet connect activated pentose phosphates continuously to nucleoside triphosphates, RNA, or a differentiated nucleotide economy. The functional specialization described here should therefore be regarded as a plausible outcome of catalyst–carrier coevolution, not as a direct inference from the distribution of nucleotide functions in modern metabolism.

### 7.6. Stage VI: Chemiosmotic Regeneration and Consolidation of the ATP Economy

The emergence of selectively permeable compartments would have converted ion gradients from transient environmental features into reusable sources of chemical work (Figure 7). Membrane-bound pyrophosphatases provide a plausible bridge to this form of bioenergetics because they couple pyrophosphate hydrolysis directly to the translocation of H^+^ or Na^+^. Their broad distribution among prokaryotes, together with phylogenetic evidence that Na^+^-translocating forms preceded the more specialized H^+^ pumps, is consistent with an early role for pyrophosphate-dependent ion transport, although it does not establish when these enzymes first arose [96].

Importantly, the coupling between pyrophosphate chemistry and an electrochemical gradient is not restricted to the hydrolytic direction. In *Rhodospirillum rubrum* chromatophores, a proton-motive force generated by the reverse energy-linked transhydrogenase reaction drove the condensation of orthophosphate to pyrophosphate [149]. Purification and reconstitution of the DCCD-sensitive H^+^-translocating pyrophosphate enzyme from the same organism provided a biochemical basis for this membrane-associated coupling [150]. Together with light-driven pyrophosphate synthesis in bacterial chromatophores, these observations establish that ion-gradient energy can, under suitable conditions, be conserved in a phosphoanhydride bond [151]. They do not demonstrate that ancestral membrane pyrophosphatases operated reversibly, but they provide an extant proof of principle for the proposed transition.

A mixed pyrophosphate–ATP bioenergetic system could then have emerged without an abrupt change in coupling principle. In reconstituted liposomes, pyrophosphate hydrolysis by a membrane pyrophosphatase generates a proton gradient that drives ATP synthesis through an F_0_F_1_ complex [147]. Pyrophosphate and ATP could therefore have been connected through a shared electrochemical intermediate: pyrophosphate hydrolysis generated the gradient, whereas ATP synthase converted that gradient into a chemically versatile nucleotide triphosphate. This arrangement would have allowed ATP-dependent chemistry to expand before ATP regeneration became the dominant source of membrane-coupled chemical energy.

Once rotary ATP synthases and nucleotide-selective catalysts became established, chemiosmosis offered a continuous and regulatable route to ATP regeneration. ATP could then centralize reactions that had previously relied on pyrophosphate, acyl and enol phosphates, or direct substrate-level phosphorylation. The transition was unlikely to have been a simple replacement. Membrane pyrophosphatases, pyrophosphate-dependent kinases, acyl phosphates, and ATP-dependent reactions could have remained functionally coupled over an extended interval, with selection progressively favouring networks that channelled diverse energy inputs into a common adenylate pool. ATP-centred bioenergetics would thus have emerged through consolidation of an existing ion-coupled phosphate economy rather than through the invention of an entirely new energetic principle.

### 7.7. Coexistence, Functional Retention, and Evolutionary Continuity

The consolidation of ATP-centred metabolism did not render pyrophosphate obsolete. Instead, pyrophosphate was retained in reactions where its formation or consumption provides a specific kinetic or thermodynamic advantage (Figure 7). Cleavage of ATP to AMP and pyrophosphate generates activated intermediates in biosynthesis, whereas removal of pyrophosphate restricts the reverse reaction and commits flux towards product formation. This principle is particularly evident in nucleic acid polymerization, where pyrophosphate release—and, in some polymerases, its subsequent hydrolysis within the catalytic complex—reinforces forward synthesis [21,41]. Pyrophosphate therefore became less important as a freely transferable energy source than as a product whose controlled removal imposes directionality.

Other pyrophosphate-dependent functions persisted alongside the adenylate economy. Specialized kinases retained the capacity to use pyrophosphate as a phosphoryl donor, while membrane pyrophosphatases continued to couple its hydrolysis to H^+^ or Na^+^ transport [18,20]. These systems preserve distinct uses of the same phosphoanhydride chemistry: direct phosphoryl transfer in one case and conversion into electrochemical work in the other. Their persistence is compatible with a prolonged period in which pyrophosphate- and ATP-dependent reactions coexisted, although the distribution of modern enzymes cannot by itself establish their order of emergence.

Pyrophosphate also acquired functions beyond intracellular energy coupling. In extracellular fluids, it inhibits calcium-phosphate crystal growth and thereby opposes inappropriate mineral deposition [100]. This role illustrates how a compound initially considered in terms of phosphate activation can acquire a chemically distinct role determined by its affinity for mineral surfaces.

The evolutionary significance of pyrophosphate therefore lies in its contextual versatility. Modern cells do not use it as a universal energy currency, but repeatedly exploit its formation, hydrolysis, or transfer to control reaction commitment, polymerization, phosphoryl flux, ion transport, and mineralization. This functional breadth is consistent with continuity from an earlier phosphate-based energetic architecture, but should not be treated as evidence of direct ancestry. Pyrophosphate is neither simply an energy donor nor merely a metabolic by-product; its role is defined by the reaction in which it is produced, consumed, or maintained.

## 8. Experimental Tests and Falsifiable Predictions

The evolutionary sequence proposed here should be regarded as a chemically testable model rather than as a direct reconstruction of a unique historical pathway. Its central transitions can be examined separately under controlled experimental conditions and subsequently combined in coupled systems. Support for the model would therefore require more than the demonstration of isolated reactions: it would depend on reconstructing the proposed links between pyrophosphate capture, phosphate-chain extension, group-transfer chemistry, and increased network persistence. Conversely, systematic failure of any of these transitions across a broad and chemically justified parameter space would constrain or weaken the corresponding stage of the model.

### 8.1. Direct Capture of Pyrophosphate by Formose-Derived Sugars

The model is defined by a series of experimentally separable transitions. The first concerns the direct entry of pyrophosphate into carbohydrate chemistry. Under conditions that lower water activity and favour substrate organization, pyrophosphate should phosphorylate selected terminal hydroxyl groups within formose-derived polyols and sugars. Experiments should therefore test chemically defined substrates and unfractionated formose mixtures across controlled mineral, metal-ion, and hydration regimes. Product identification by ^31^P NMR, isotope tracing, and tandem mass spectrometry would establish whether phosphate transfer is regioselective and whether terminal products accumulate above hydrolysis and orthophosphate controls. Existing studies define plausible boundary conditions for this reaction but do not yet demonstrate the proposed transformation itself [16,64].

### 8.2. Formation of Pentose Diphosphates and Triphosphates

The second prediction is that activated products of carbon or sulfur chemistry can extend pentose phosphate monoesters into compounds containing P–O–P bonds. Enol and acyl phosphates should therefore be tested as donors for the stepwise conversion of ribose and other pentose monophosphates into chemically defined diphosphate or triphosphate derivatives. This experiment must distinguish phosphate-chain extension from the addition of independent monoester groups at other hydroxyl positions. The prebiotic accessibility and phosphoryl-transfer capacity of phosphoenolpyruvate and acetyl phosphate provide a basis for this test, but neither has yet been shown to produce pentose diphosphates and triphosphates through a continuous enzyme-free sequence [76,84].

### 8.3. Group-Transfer Activity of Activated Pentoses

The third prediction concerns function. Once isolated, activated pentose diphosphates and triphosphates should promote chemically defined group-transfer reactions involving nucleobases, amino acids, glycerol phosphates, or fatty acids. Product formation should be accompanied by the leaving group expected from the structure of the donor, including pyrophosphate in phosphoribosyl-transfer reactions. PRPP-mediated AMP formation on silica establishes a direct precedent for phosphoribosyl transfer, whereas energy-dissipative acylation shows that activated chemistry can be coupled to the production and selection of membrane-forming amphiphiles [134]. These observations support the feasibility of the reaction classes, but not the proposed role of pentose diphosphates and triphosphates as common carriers.

### 8.4. Integration, Persistence, and Partial Recycling

The strongest test would integrate these reactions rather than examine them in isolation. Coupled systems operating within mineral pores, regulated wet–dry cycles, or primitive vesicles should sustain activated intermediates for longer, increase cumulative product formation, or permit partial recycling of pyrophosphate relative to matched uncoupled controls. Persistence should be assessed across repeated cycles rather than from maximum yield in a single reaction. Wet–dry environments can regulate condensation and hydrolysis, while mixed amphiphiles can form stable compartments under geochemically relevant conditions, providing experimentally tractable settings for such integration [32,137].

No individual positive result would identify a unique historical route. Conversely, systematic failure of one transition across a broad and chemically justified parameter space would constrain or eliminate that part of the sequence. The model would gain support only if its central couplings can be reconstructed: capture of pyrophosphate by carbon compounds, regeneration of activated phosphate within the network, incorporation of molecular carriers, and increased persistence when these processes are combined.

## 9. Conclusions

Inorganic pyrophosphate may have provided a chemically simple transitional link between geochemical phosphate activation and the emergence of nucleotide-based bioenergetics. Its plausible prebiotic formation, capacity for phosphoryl transfer, and potential regeneration from activated phosphate compounds support a role as a recurrent local coupling intermediate rather than as a universal energy currency.

The strongest elements of this model are supported by direct experimental precedents, whereas pyrophosphate-dependent phosphorylation of formose-derived sugars, partial energetic closure within coupled reaction networks, and the proposed role of activated pentose diphosphates and triphosphates as molecular carriers remain hypothetical. ATP-centred bioenergetics may therefore have emerged by consolidating functions previously distributed among pyrophosphate, activated phosphate compounds, phosphorylated carbon intermediates, and ion gradients. The value of the model lies in the fact that these proposed transitions are chemically constrained and experimentally testable.

Even successful reconstruction of these energetic and chemical couplings would not, by itself, explain the emergence of a self-sustaining living system. The origins of heredity, information-bearing polymers, catalytic specificity, and coordinated biological regulation lie beyond the scope of the present hypothesis and would require additional transitions not addressed here.

The model will be strengthened not by accumulating additional isolated chemical precedents, but by reconstructing coupled systems in which pyrophosphate capture, regeneration, and product persistence operate together under geochemically plausible conditions.

## Figures and Tables

**Table 1:** Relevant thermodynamic data for sugar-phosphate hydrolysis.

Hydrolysis Reaction	ΔG°′ (kJ mol^−1^)	Ref.
α-D-ribose-1-phosphate + H_2_O → D-ribose + P_i_	≈−22.6	[43]
D-ribose-5-phosphate + H_2_O → D-ribose + P_i_	≈−15.0	[42]
D-ribulose-5-phosphate + H_2_O → D-ribulose + P_i_	≈−13.4	[42]
α-D-glucose-1-phosphate + H_2_O → D-glucose + P_i_	≈−20.8	[44,45]
D-glucose-6-phosphate + H_2_O → D-glucose + P_i_	≈−13.7	[44]
D-fructose-6-phosphate + H_2_O → D-fructose + P_i_	≈−13.7	[42]
D-fructose-1-phosphate + H_2_O → D-fructose + P_i_	≈−10.3	[46,47]
PRPP + H_2_O → ribose-5-phosphate + PP_i_	≈−29.3	[48]

**Table 2:** Experimentally supported hydrolysis energetics of candidate phosphate donors.

Hydrolysis Reaction	ΔG°′ (kJ mol^−1^)	Ref.
Phosphoenolpyruvate + H_2_O → pyruvate + P_i_	≈−61.9	[69]
Carbamoyl phosphate + H_2_O → carbamate + P_i_	≈−51	[70]
1,3-BPG + H_2_O → 3-phosphoglycerate + P_i_	≈−47.7	[71]
Phosphocreatine + H_2_O → creatine + P_i_	≈−44	[72,73]
Acetyl phosphate + H_2_O → acetate + P_i_	≈−42.7	[74,75]

## Data Availability

No new data were created or analyzed in this study. Data sharing is not applicable to this article.

## Supplementary Materials

The following supporting information can be downloaded at https://www.mdpi.com/article/10.3390/biology15181592/s1, Table S1: Known Reactions That Release Inorganic Pyrophosphate (PP_i_).

## Abbreviations

1,3-BPG	1,3-bisphosphoglycerate
ACP	acyl carrier protein
ADP	adenosine diphosphate
AMP	adenosine monophosphate
ATP	adenosine triphosphate
AVP1	Arabidopsis vacuolar H^+^-pyrophosphatase 1
CDP	cytidine diphosphate
CTP	cytidine triphosphate
DCCD	N,N′-dicyclohexylcarbodiimide
DNA	deoxyribonucleic acid
EC	Enzyme Commission
FAD	flavin adenine dinucleotide
G2P	glyceraldehyde 2-phosphate
G3P	glyceraldehyde 3-phosphate
HCN	hydrogen cyanide
HEPC	high-energy phosphate compound
NAD^+^	nicotinamide adenine dinucleotide
NADP^+^	nicotinamide adenine dinucleotide phosphate
NMR	nuclear magnetic resonance
PEP	phosphoenolpyruvate
P_i_	inorganic orthophosphate
PP_i_	inorganic pyrophosphate
PRPP	5-phosphoribosyl 1-pyrophosphate
RNA	ribonucleic acid
UDP	uridine diphosphate
UTP	uridine triphosphate
