# Supplementary material for: The Role of Pyrophosphate in the Origin of Life: An Energy-Based Hypothesis
Source: Biology (Basel). 2026 Sep 9;15(18):1592. doi: 10.3390/biology15181592 (PMC13604480; doi:10.3390/biology15181592)
Supplement: Supplementary file 1 [file biology-15-01592-s001.zip › biology-4504782-supplementary.pdf]

Table S1. Known Reactions That Release Inorganic Pyrophosphate (PP_i). Selected reaction classes relevant to biosynthesis, activation chemistry, and metabolism

Reaction class	General reaction	Representative examples
DNA synthesis	dNTP + DNA_n → DNA_{n+1} + **PPi**	DNA polymerases (EC 2.7.7.7) in replication and repair
RNA synthesis	NTP + RNA_n → RNA_{n+1} + **PPi**	RNA polymerases (EC 2.7.7.6); DnaG primases (EC 2.7.7.101); AEP primases (EC 2.7.7.102)
Amino acid activation for translation	Amino acid + ATP + tRNA → aminoacyl-tRNA + AMP + **PPi**	Aminoacyl-tRNA synthetases (EC 6.1.1.1–6.1.1.27)
Fatty acid activation	Fatty acid + CoA + ATP → acyl-CoA + AMP + **PPi**	Acyl-CoA synthetases (EC 6.2.1.-); long-chain-fatty-acid–CoA ligase (EC 6.2.1.3)
Non-ribosomal peptide activation	Carboxylate substrate + ATP → acyl-AMP intermediate + **PPi**	NRPS adenylation domains / carrier-protein ligases (EC 6.2.1.-)
Ubiquitin and ubiquitin-like activation	Ubiquitin (or UBL) + ATP + E1 → Ub-AMP intermediate + **PPi**	Ubiquitin E1 (EC 6.2.1.45); SUMO E1 and NEDD8 E1 (EC 6.2.1.-)
Activated sugar donor formation	Sugar-1-P + NTP → NDP-sugar + **PPi**	UDP-glucose pyrophosphorylase (EC 2.7.7.9); ADP-glucose pyrophosphorylase (EC 2.7.7.27); GDP-mannose pyrophosphorylase (EC 2.7.7.13/2.7.7.22)
UDP-glucose formation	Glucose-1-phosphate + UTP → UDP-glucose + **PPi**	UDP-glucose pyrophosphorylase (EC 2.7.7.9)
Nucleotide salvage / phosphoribosyl transfer	Base + PRPP → nucleotide monophosphate + **PPi**	HGPRT (EC 2.4.2.8); APRT (EC 2.4.2.7); orotate phosphoribosyltransferase (EC 2.4.2.10)
De novo purine entry step	PRPP + glutamine → phosphoribosylamine + glutamate + **PPi**	Amidophosphoribosyltransferase (EC 2.4.2.14)
Other PRPP-dependent biosyntheses	PRPP + acceptor → phosphoribosylated product + **PPi**	ATP phosphoribosyltransferase (EC 2.4.2.17) in histidine biosynthesis; anthranilate phosphoribosyltransferase (EC 2.4.2.18) in tryptophan biosynthesis; nicotinate-nucleotide diphosphorylase (EC 2.4.2.19) in NAD biosynthesis
Sulfate activation	Sulfate + ATP → APS + **PPi**	ATP sulfurylase (EC 2.7.7.4)
Coenzyme A biosynthesis	Phosphopantetheine + ATP → dephospho-CoA + **PPi**	Phosphopantetheine adenylyltransferase (EC 2.7.7.3)

NAD biosynthesis / adenylyl transfer	NMN or NaMN + ATP → NAD or deamido-NAD + **PPi**	NMN adenylyltransferase (EC 2.7.7.1); NaMN adenylyltransferase (EC 2.7.7.18)
FAD biosynthesis	FMN + ATP → FAD + **PPi**	FAD synthetase (EC 2.7.7.2)
Isoprenoid-chain elongation	Allylic prenyl diphosphate + IPP → elongated prenyl diphosphate + **PPi**	Geranyl diphosphate synthase (EC 2.5.1.1); farnesyl diphosphate synthase (EC 2.5.1.10); geranylgeranyl diphosphate synthase (EC 2.5.1.29); long-chain polyprenyl diphosphate synthases (EC 2.5.1.-)
CDP-diacylglycerol formation	Phosphatidic acid + CTP → CDP-diacylglycerol + **PPi**	CDP-diacylglycerol synthase, CdsA/CDS1/CDS2 (EC 2.7.7.41)
CDP-choline formation	Phosphocholine + CTP → CDP-choline + **PPi**	CTP:phosphocholine cytidylyltransferase (EC 2.7.7.15)
CDP-ethanolamine formation	Phosphoethanolamine + CTP → CDP-ethanolamine + **PPi**	CTP:phosphoethanolamine cytidylyltransferase (EC 2.7.7.14)
